# Modelling the delay between pharmacokinetics and EEG effects of morphine in rats: binding kinetic versus effect compartment models

**DOI:** 10.1007/s10928-018-9593-x

**Published:** 2018-05-18

**Authors:** Wilhelmus E. A. de Witte, Vivi Rottschäfer, Meindert Danhof, Piet H. van der Graaf, Lambertus A. Peletier, Elizabeth C. M. de Lange

**Affiliations:** 1 Division of Pharmacology, Leiden Academic Centre for Drug Research, Leiden University, 2333 CC Leiden, The Netherlands; 2 Mathematical Institute, Leiden University, 2333 CA Leiden, The Netherlands; 3 Certara Quantitative Systems Pharmacology, Canterbury Innovation Centre, Canterbury, CT2 7FG UK

**Keywords:** Drug–target binding kinetics, Effect compartment model, PKPD modelling, Hysteresis, Morphine

## Abstract

Drug–target binding kinetics (as determined by association and dissociation rate constants, *k*_*on*_ and *k*_*off*_) can be an important determinant of the kinetics of drug action. However, the effect compartment model is used most frequently instead of a target binding model to describe hysteresis. Here we investigate when the drug–target binding model should be used in lieu of the effect compartment model. The utility of the effect compartment (EC), the target binding kinetics (TB) and the combined effect compartment–target binding kinetics (EC–TB) model were tested on either plasma (EC_PL_, TB_PL_ and EC–TB_PL_) or brain extracellular fluid (ECF) (EC_ECF_, TB_ECF_ and EC–TB_ECF_) morphine concentrations and EEG amplitude in rats. It was also analyzed when a significant shift in the time to maximal target occupancy (Tmax_TO_) with increasing dose, the discriminating feature between the TB and EC model, occurs in the TB model. All TB models assumed a linear relationship between target occupancy and drug effect on the EEG amplitude. All three model types performed similarly in describing the morphine pharmacodynamics data, although the EC model provided the best statistical result. The analysis of the shift in Tmax_TO_ (∆Tmax_TO_) as a result of increasing dose revealed that ∆Tmax_TO_ is decreasing towards zero if the *k*_*off*_ is much smaller than the elimination rate constant or if the target concentration is larger than the initial morphine concentration. The results for the morphine PKPD modelling and the analysis of ∆Tmax_TO_ indicate that the EC and TB models do not necessarily lead to different drug effect versus time curves for different doses if a delay between drug concentrations and drug effect (hysteresis) is described. Drawing mechanistic conclusions from successfully fitting one of these two models should therefore be avoided. Since the TB model can be informed by in vitro measurements of *k*_*on*_ and *k*_*off*_, a target binding model should be considered more often for mechanistic modelling purposes.

**Electronic supplementary material:**

The online version of this article (10.1007/s10928-018-9593-x) contains supplementary material, which is available to authorized users.

## Introduction

Drug–target binding kinetics is an important criterion in the selection of drug candidates, as it can be a determinant of the time course and the selectivity of drug effect [1–4].

However, the in vivo time course of drug action is influenced by multiple factors including plasma pharmacokinetics, target site distribution, target binding kinetics, competition with endogenous ligands, turnover of the target, signal transduction kinetics and the kinetics of homeostatic feedback. As a consequence, the influence of binding kinetics on drug action can only be understood in conjunction with these kinetic processes and its relevance is still not fully understood and subject to an ongoing debate [3, 5–8].

One of the arguments against an important role of binding kinetics for in vivo drug action is that binding kinetics are most often not required to get a good fitting PKPD model for small molecules. However, numerous examples are available were binding kinetic models have been successfully applied, and binding kinetics are routinely incorporated in models for biologics and PET data [9–17]. The sparsity of target binding PKPD models for small molecules can be explained by the relatively fast binding kinetics of many drugs currently on the market, compared to their pharmacokinetics [3]. In addition, when a delay between drug concentrations and effect is observed, this delay is often described by an effect compartment or indirect response model [18, 19].

Here we study the difference between the effect compartment (EC) model, the target binding (TB) model, the direct effect (DE) and the indirect effect (IE) model, which are described below. The EC model describes the delay between pharmacokinetics (PK) and pharmacodynamics (PD) by including first order distribution of the drug into and out of a hypothetical target-site (biophase) compartment, which drives the pharmacodynamics [20]. The indirect effect (IE) model describes the delay between pharmacokinetics and pharmacodynamics by the zero order synthesis and first order degradation of an effector molecule which represents the pharmacodynamics [21]. The target binding (TB) model describes the delay between pharmacokinetics and pharmacodynamics by the second order drug–target association and first order dissociation of the drug–target complex [22–24]. The DE model describes no delay between the pharmacokinetics and pharmacodynamics and links the drug concentration directly to the effect measurements. All of these models can account for saturation of the PK–PD relationship (i.e. increasing drug concentrations do not increase the drug effect beyond a certain concentration), but this saturation can occur at different steps in the process between the drug concentration and the drug effect. For the EC model, the relationship between drug concentrations in the effect compartment and the effect can be saturable. For the IE model, both the relationship between the drug concentration and the synthesis or degradation rate of the effector molecule can be saturable. For the TB model, the relationship between the drug concentration and the target occupancy is saturable and the relationship between target occupancy and effect can be saturable. For the DE model, the relationship between the drug concentration and the drug effect can be saturable.

These models thus result in a zero, first and second order formation of the the pharmacodynamics in the EC, TB and IE model, respectively. This results in different dose dependencies of the time to the maximal effect (Tmax_PD_). As a current paradigm, the shift in Tmax_PD_ (∆Tmax_PD_) in a PKPD dataset as a consequence of a change in the dose identifies the appropriate PKPD model to describe the data: with increasing dose, the Tmax_PD_ can increase for the indirect response model, decrease for the TB model and is constant for the EC model [25–27].

However, in contrast to common belief, the indirect response model does not always result in an increasing Tmax_PD_ with increasing doses but can also give rise to a decreasing Tmax_PD_ with increasing doses, as shown by Peletier et al. [28]. If the relationship between target occupancy and effect is not delayed and monodirectional (i.e. an increase in target occupancy never results in a decrease in effect and vice versa), the Tmax_PD_ coincides with the time to the maximal target occupancy (Tmax_TO_). To start with this most simple situation, we focus in this paper only on the Tmax_TO_. A comprehensive analysis of the conditions for which a shift in Tmax_TO_ for changing doses occurs in a TB model is currently not available. It might be that EC models have been used while TB models could have been applied equally well to describe the data in previous PKPD studies.

One example in which performance of TB and EC models has been investigated indicates comparable performance in describing the data of eight calcium channel blockers, but this study used only one dose level for all drugs [14] and therefore cannot be used to validate the relationship between dose and ∆Tmax_PD_. An additional complexity in choosing the most appropriate PKPD model to describe PKPD data is that, for most drugs, factors as target site distribution, drug–target binding and turnover of signaling molecules occur in parallel. It is not always needed to incorporate all these factors in the PKPD model, as only the rate limiting mechanism is required for a proper model fit that describes the observed data. However, leaving out such factors will never lead to understanding of the individual contributions and the interplay between these factors. Combined EC–TB models [13, 29, 30] as well as combined IE–TB models [10] have been applied successfully to discriminate between the contributions of separate factors. However, this discrimination is not always possible if one of the factors is relatively fast and does not contribute significantly to the delay between pharmacokinetics and pharmacodynamics [31–33]. In short, the relevance of drug–target binding kinetics cannot be excluded if one of the other models is successfully fitted to a dataset, and there is a need to generate more insight into the difference between the TB model and the EC model.

The aim of the current study is to investigate if the TB and EC model can give similar drug effect versus time curves and under what conditions this will occur. In this study, a historical PKPD dataset for morphine was used [34] to compare the goodness of fit for the TB model with the EC model and the combined EC–TB model in describing the time course of the EEG effect following administration of three different doses of morphine (4, 10 and 40 mg/kg). Both plasma and brain extracellular fluid (ECF) drug concentrations were measured and tested in this study to be connected to the pharmacodynamics via an EC, TB or EC–TB model. Subsequently, a more general insight in the shift of Tmax_TO_ (∆Tmax _TO_) for different dose levels in the drug–target binding model is obtained to identify for what parameter values the TB model can be discriminated from the EC model based on the ∆Tmax_TO_. To that end, comprehensive simulations and mathematical model analysis was performed for a wide range of drug–target association and dissociation rate constants, for various plasma elimination rate constants, target concentrations, and dose levels.

## Methods

### Pharmacokinetic and pharmacodynamic (PKPD) data of morphine in rats

All pharmacokinetics and pharmacodynamics data used in this study were obtained from the experiments described earlier [35]. In short: Morphine was intravenously administered to Male Wistar rats, during a 10-min infusion, in 4 different dose groups: 0, 4, 10 or 40 mg/kg with 5, 29, 11 and 14 animals, respectively. The P-glycoprotein (Pgp) inhibitor GF120918 or vehicle was given as a continuous infusion. In the group of 29 animals that received 4 mg/kg morphine, 9 animals received GF120918, the other 20 animals received the vehicle. Furthermore, while plasma concentrations were measured in all animals, brain ECF concentrations were measured with microdialysis in 29 animals, of which 15 received 4 mg/kg, 0 received 10 mg/kg, 9 received 40 mg/kg and 5 received 0 mg/kg morphine, see also Table 1.

**Table 1:** Overview of treatment groups and data collection

Morphine dose	Pgp inhibitor	Plasma pharmacokinetics data	ECF pharmacokinetics data	EEG data	Dosed animals
0 mg/kg	−	0	5	5	5
4 mg/kg	−	20	7	16	20
4 mg/kg	+	9	8	9	9
10 mg/kg	−	11	0	11	11
40 mg/kg	−	14	9	13	14
Total		54	29	54	59

For the modelling data set, all data entries without time recordings, without concentration data or with concentration data equal to 0 were removed from the dataset. The lower limit of quantification for morphine in plasma samples was 88 nM and 1.75 nM for morphine in ECF samples. The pharmacodynamics of morphine was measured as the amplitude in the δ frequency range (0.5–4.5 Hz) of the EEG and recorded every minute. The EEG data were further averaged for every 3-min interval to reduce the noise and decrease the model fitting time.

### General model fitting methods

Data fitting was based on minimization of the objective function value (OFV = − 2 * log likelihood) as implemented in NONMEM 7.3 [36]. Simulations and visualisations were performed in RStudio version 3.4.0. To account for the number of parameters for the comparison of non-nested models, the Akaike Information Criterion (AIC) was calculated by adding two times the number of estimated parameters to the OFV [37]. Variability in the data was described by IIV (Inter Individual Variability: variability in parameter values between animals) and a residual error term. IIV was implemented assuming a log-normal distribution according to Eq. 1:

1 $$ P_{i} = P_{pop} \,*\, e^{{\eta_{i} }} $$

In which *P*_*i*_ is the individual parameter value, *P*_*pop*_ is the typical parameter value in the population and *η*_*i*_ is normally distributed around a mean of zero with variance *ω*^2^ according to Eq. 2:

2 $$ \eta_{i} \sim N\left( {0, \omega^{2} } \right) $$

The remaining variation between the data and the model predictions are incorporated as residual error for which both a proportional (Eq. 3) and a combined proportional and additive (Eq. 4) error model were tested.

3 $$ obs_{ij} = pred_{ij} *\left( {1 + \varepsilon_{prop,ij} } \right) $$

4 $$ obs_{ij} = pred_{ij} \,*\,\left( {1 + \varepsilon_{prop,ij} } \right) + \varepsilon_{add,ij} $$

In these equations, *obs*_*ij*_ is the observation, *pred*_*ij*_ is the model prediction, *ε*_*prop,ij*_ is the proportional error and *ε*_*add,ij*_ is the additive error for individual *i* at time point *j*. Both *ε*_*prop,ij*_ and *ε*_*add,ij*_ are normally distributed around a mean of zero with variance σ^2^ according to Eqs. 5 and 6:

5 $$ \varepsilon_{prop,ij} \sim N\left( {0,\sigma^{2} prop} \right) $$

6 $$ \varepsilon_{add,ij} \sim N\left( {0,\sigma^{2} add} \right). $$

### Morphine plasma pharmacokinetics modelling

One-compartment, two-compartment and three-compartment models were fitted to the plasma pharmacokinetics data, with both proportional and additive plus proportional error models, and with IIV on the various parameters. The differential equations and model scheme are given in Supplement S1. The best fits (based on AICs) of each structural model were compared for their Goodness Of Fits (GOFs) and AICs. Since the purpose of the plasma pharmacokinetics modelling was to get the best possible input for the pharmacodynamics modelling, GOF was assessed by the AIC and by individual fits. Over- or underestimation of IIV and population parameter estimates and high uncertainties in population parameter estimates were not regarded as problematic, since only the right individual parameter estimates were required for pharmacodynamics modeling.

### Morphine brain ECF pharmacokinetics Modelling

The individual parameter estimates that were estimated to describe the plasma pharmacokinetics were used as fixed parameters to describe the plasma pharmacokinetics profile as input for the brain ECF concentrations. To describe the ECF concentrations, we thus assumed that the distribution of the drug into and out of the ECF did not lead to a change in plasma concentrations. The differential equations and model scheme are given in Supplement S1. The best fits, based on the AICs, of each structural model were compared for their GOFs and AICs. Since the purpose of the brain ECF pharmacokinetics modelling was to get the best possible input for the pharmacodynamics modelling, GOF was assessed by the AIC and by individual fits. Over- or underestimation of IIV and population parameter estimates and high uncertainties in population parameter estimates were not regarded as problematic, since only the right individual parameter estimates were required for pharmacodynamics modeling.

### EEG pharmacodynamics modelling

To maximize the identifiability of the pharmacodynamics model parameters, all pharmacokinetic parameters were used as fixed parameters to describe the plasma and brain ECF concentrations as input for all the described pharmacodynamics models to describe EEG effects [38]. The different type of models that were tested are outlined in Table 2. The differential equations are given in Supplement S1. For each model, the most informative variations on the model structure are given in the results section.

**Table 2:** Overview of the different model types, the data that were used and the model numbers as used in this manuscript

Model type	Concentrations linked to effect	Model number
EC	Plasma	EC_PL_1 – EC_PL_4
EC	ECF	EC_ECF_1
TB	Plasma	TB_PL_1–TB_PL_5
TB	ECF	TB_ECF_1
EC–TB	Plasma	ECTB_PL_1–ECTB_PL_5
IE	ECF	IE_ECF_1
DE	ECF	DE_ECF_1

*EC* effect compartment, *TB* target binding, *EC*–*TB* effect compartment–target binding, *IE* indirect effect, *DE* direct effect, *ECF* brain extracellular fluid

To compare structural models that linked plasma or brain ECF concentrations directly to the PD, the models that used plasma pharmacokinetics were fitted to the reduced dataset that only contained animals with plasma PK, brain ECF and EEG measurements. Model comparison was based on the AIC, visual inspection of the GOF and a VPC (Visual Predictive Check) to check if the IIV was captured appropriately.

### Drug–target binding model simulations

Simulations with a one-compartment binding model with IV administration were performed for a wide range of *k*_*on*_ and *k*_*off*_ values and for a variety of elimination rate constants, target concentrations and drug dose levels (Table 3). The Tmax_TO_ was compared for two different doses to determine the influence of the drug dose on the Tmax_TO_. The ∆Tmax_TO_ values were calculated by subtracting the Tmax_TO_ of the highest dose from the Tmax_TO_ of the lowest dose and ∆Tmax_TO_ was plotted against *k*_*on*_ and *k*_*off*_.

**Table 3:** Overview of the different simulation scenarios

Simulation #	*k*_*el*_ (1/h)	*R*_*tot*_ (nM)	*C*_0_ low (* *K*_*D*_)	C_0_ high (* *K*_*D*_)
1	0.03	0.1	0.5	5
2	0.03	0.1	5	50
3	0.03	10	0.5	5
4	0.3	0.1	0.5	5

For each simulation, *k*_*on*_ and *k*_*off*_ varied between 10^−3^ and 10^3^/nM/h and between 10^−3^ and 10^3^ h^−1^, respectively

## Results

### Morphine pharmacokinetics modelling

Modelling of morphine pharmacokinetic data in plasma and brain ECF as described in Supplement S1 identified very similar model structures as previously described for pharmacokinetic modelling of the same dataset by Groenendaal and coworkers [35]. In short, the plasma concentrations were described by a three-compartment model and the ECF concentrations were described by passive distribution into and out of the brain combined with saturable active influx and first-order efflux.

### EEG pharmacodynamics modelling

#### EC_PL_ model fitting

**Fig. 1:**
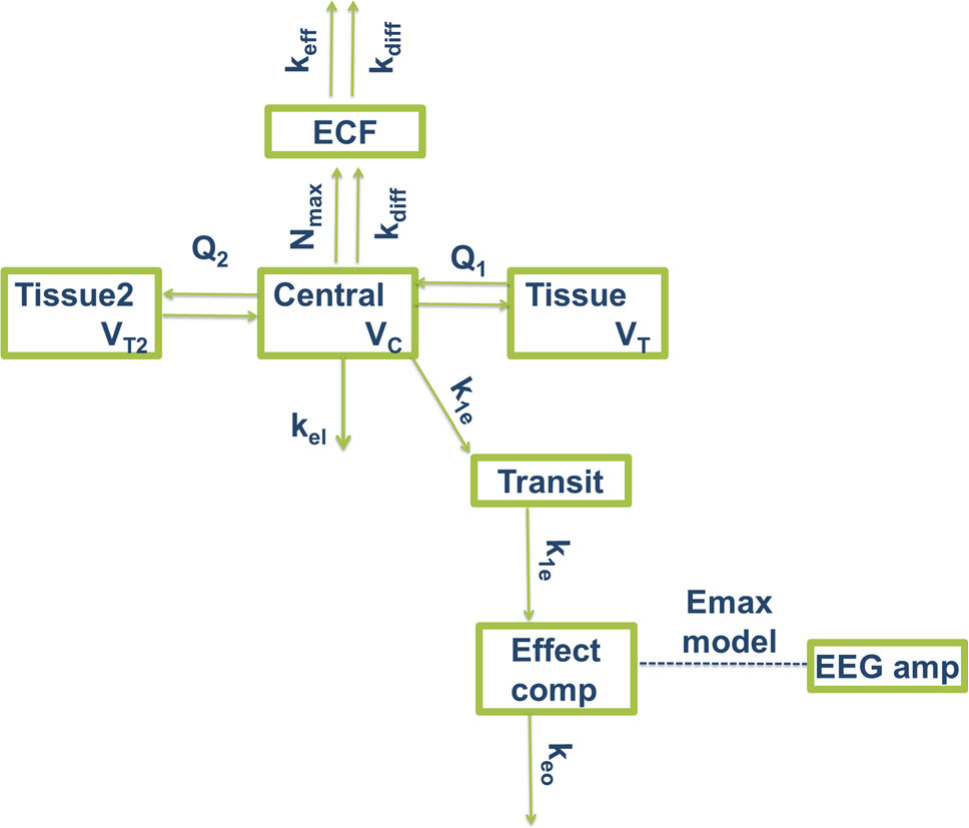
Schematic representation of the EC_PL_ model structure that was used to describe the morphine EEG amplitudes over time. *k*_*ie*_, first-order in- and outward distribution rate constant for the transit compartment. *k*_*eo*_, first-order outward distribution rate constant from the effect compartment. The effect compartment concentrations were linked to the EEG amplitude by a sigmoidal Emax model. The distribution from plasma to the tissue compartments and the brain ECF compartment is described in Supplement S1. The arrows indicate morphine flows, the dotted line indicates a direct relationship

EC and TB models have been applied to the morphine data to describe the relationship between the observed plasma concentrations and EEG amplitude and direct effect (DE), indirect effect (IE), EC and TB models have been applied to brain ECF and EEG amplitude data. The differential equations for these models are given in Supplement S1. Firstly, the originally published EC_PL_ model structure was optimized by adding a slope-parameter, which describes the linear decline of EEG amplitude over time during the experiment independently of the drug effect, and by including IIV on the baseline EEG amplitude only. For this model, a transit compartment was required between the plasma and the effect compartment for the best description of the data [34]. An overview of the different variations on this basic model structure is given in Table 4. The structure of all EC_PL_ is identical and is depicted in Fig. 1. Based on the AIC, the parameter estimates and the GOF, model EC_PL_1 was chosen as the best parameterization for the effect compartment model in Fig. 1.

**Table 4:** Parameter values and objective function values of the tested EC models describing the EEG data, based on plasma concentrations

	Parameter definition	EC_PL_1 selected model	EC_PL_2 no slope	EC_PL_3 *k*_1*e*_ = *k*_*eo*_	EC_PL_4 no Pgp effect
OFV		44,748.0	45,084.2	44,853.3	44,868.4
AIC		44,770.0	45,104.2	44,871.3	44,886.4

Parameter	Parameter definition	Value (%CV)	Value (%CV)	Value (%CV)	Value (%CV)
*k*_1*e*_(/min)	Distribution to transit compartment	0.0393 (18)	0.0432 (10)	0.0403 (10)	0.0375 (8)
*k*_*eo*_(/min)	Distribution from effect compartment	0.0382 (14)	0.0458 (9)	–	0.0375 (8)
*k*_1*e*_–*Pgp* (/min)	Distribution to transit with Pgp blocker	0.0565 (44)	0.0661 (38)	0.0295 (18)	–
*k*_*eo*_–*Pgp* (/min)	Distribution from effect with Pgp blocker	0.016 (46)	0.0203 (20)	–	–
*E*_0_ (µV)	Baseline EEG amplitude	45.1 (4)	42.2 (4)	45.8 (4)	45.9 (4)
*E*_*max*_ (µV)	Maximal increase in EEG amplitude	27.9 (23)	25.3 (16)	26.1 (18)	27.0 (18)
*EC*_50_ (nM)	concentration giving half-maximal increase in EEG amplitude	1270 (52)	1220 (31)	912 (37)	1000 (37)
*N*_*H*_	Hill factor	1.44 (43)	2.02 (27)	1.46 (36)	1.37 (33)
*slope* (µV/min)	Increase in baseline EEG over time	− 0.024 (22)	0 FIX	− 0.0263 (15)	− 0.0267 (15)
*ω*^2^ *E*_0_ (µV)	IIV variance on *E*_0_	0.111 (20)	0.125 (19)	0.115 (20)	0.116 (20)
*σ* ^2^ *prop*	Variance of proportional error	0.0554 (7)	0.0584 (7)	0.0562 (6)	0.0564 (6)

#### TB_PL_ model fitting

**Fig. 2:**
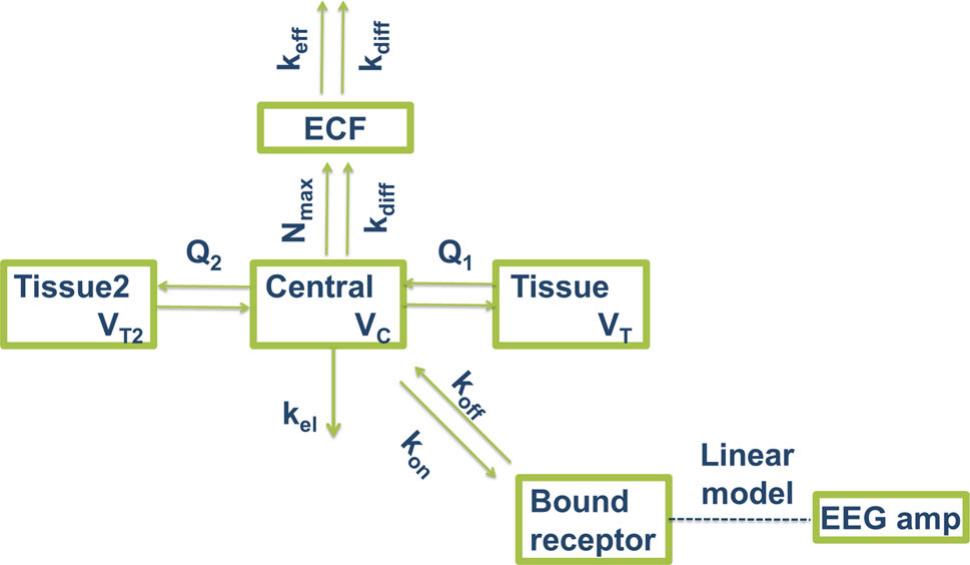
Schematic representation of the TB_PL_ model structure that was used to describe the morphine EEG amplitudes over time. *k*_*on*_ is the second-order drug-target association rate constant. *k*_*off*_ is the first-order drug-target dissociation rate constant. Target occupancy is linearly related to the EEG amplitude. The distribution from plasma to the tissue compartments and the brain ECF compartment is described in Supplement S1. The arrows indicate morphine flows, the dotted line indicates a direct relationship

The TB_PL_ model was applied to describe target binding from plasma, all TB_PL_ models in Table 5 shared the same structure as represented in Fig. 2. The parameter estimation results are given in Table 5. Since the target concentration is of influence only if it is similar to the drug concentration (which is mostly above 100 nM in plasma and in brain ECF, as shown in Supplement S1), the target concentration could not be estimated in this model and was fixed to an arbitrary low value of 1 nM in the model estimations. This low target concentration prevents the influence of the target concentration on the EEG amplitude in the model. The influence of blocking Pgp has been incorporated by estimating separate parameter values with and without the presence of Pgp blocker. While the influence of blocking Pgp on the *k*_*off*_ or *K*_*D*_ is mechanistically not plausible, the improved model fits for the models which incorporate these influences might indicate that the estimated *k*_*off*_ and *K*_*D*_ values refer to apparent values which include not only the molecular properties. The target occupancy is linearly related to the EEG amplitude in model TB_PL_1–TB_PL_5, as nonlinear relationships could not be identified accurately in this study. On basis of the objective function values, model TB_PL_4 was selected as the best drug–target binding model. It should be noted that the AIC of model TB_PL_4 is 338 points higher than model EC_PL_1, which means that model EC_PL_1 performs better in fitting the data. All TB_PL_ models have one compartment less than the transit–EC models EC_PL_1–EC_PL_4. Therefore, the combined EC–TB_PL_ models EC–TB_PL_1 to EC–TB_PL_5 were developed.

**Table 5:** Parameter values and objective function values of the tested TB_PL_ models describing the EEG data, based on plasma concentrations

	Parameter definition	TB_PL_1 no Pgp effect slope = 0	TB_PL_2 no Pgp effect	TB_PL_3 Pgp on k_off_	TB_PL_4 selected model	TB_PL_5 slope = 0
OFV		45,677.7	45,170.1	45,166.6	45,092.1	45,536.9
AIC		45,689.7	45,184.1	45,182.6	45,108.1	45,550.9

Parameter	Parameter definition	Value (%CV)	Value (%CV)	Value (%CV)	Value (%CV)	Value (%CV)
*k*_*off*_ (/min)	Dissociation rate constant	0.017 (8)	0.0103 (13)	0.0109 (17)	0.009 (26)	0.0149 (15)
*k*_*off*_–*Pgp* (/min)	Dissociation rate constant with Pgp blocker	–	–	0.0087 (26)	–	–
*K*_*D*_ (nM)	Equilibrium constant	1980 (37)	995 (36)	935 (37)	1570 (59)	3610
*K*_*D*_–*Pgp* (nM)	Equilibrium constant with Pgp blocker				381 (88)	715
*E*_0_ (µV)	Baseline EEG amplitude	42.4 (4)	45.9 (4)	45.8 (4)	45.4 (4)	42.2
*E*_*max*_ (µV)	Maximal increase in EEG amplitude	32.2 (14)	29.3 (13)	28.9 (13)	32.9 (20)	38.9
*R*_*tot*_ (nM)	Target concentration	1 FIX	1 FIX	1 FIX	1 FIX	1 FIX
*slope* (µV/min)	Increase in baseline EEG over time	0 FIX	− 0.0313 (13)	− 0.0315 (12)	− 0.0299 (12)	0 FIX
*ω*^2^ *E*_0_ (µV)	IIV variance on *E*_0_	0.135 (18)	0.117 (20)	0.117 (20)	0.113 (19)	0.13 (17)
*σ* ^2^ *prop*	Variance of proportional error	0.0639 (6)	0.059 (6)	0.059 (6)	0.0584 (6)	0.0626 (6)

#### EC–TB_PL_ model fitting

**Fig. 3:**
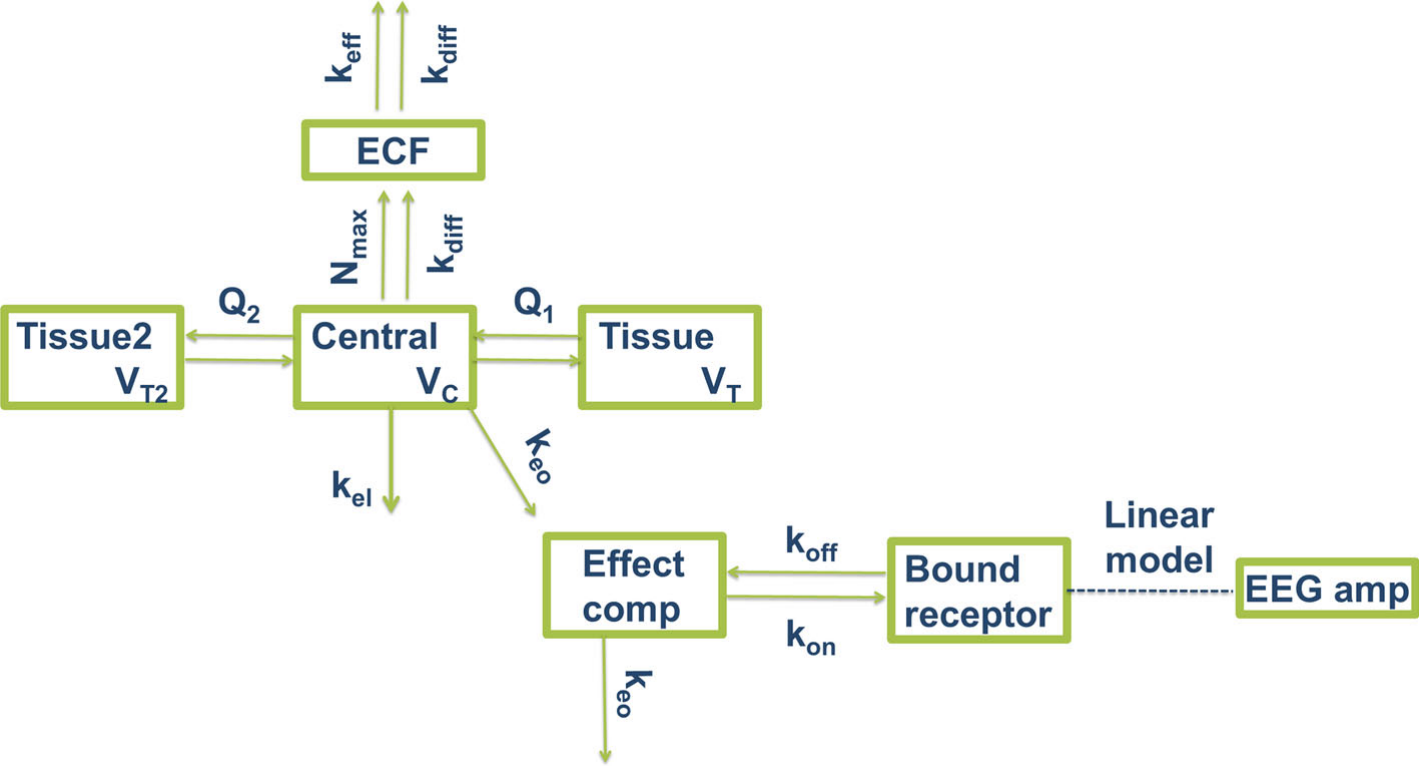
Schematic representation of the EC–TB_PL_ model structure that was used to describe the morphine EEG amplitudes over time. *k*_*on*_ is the second-order drug-target association rate constant. *k*_*off*_ is the first-order drug-target dissociation rate constant. *k*_*eo*_ is the first-order distribution rate constant into and out of the effect compartment. Target occupancy is linearly related to the EEG amplitude. The distribution from plasma to the tissue compartments and the brain ECF compartment is described in Supplement S1. The arrows indicate morphine flows, the dotted line indicates a direct relationship

The EC–TB_PL_ model structure that was tested to describe the EEG data is shown in Fig. 3. This model enables the comparison between the target binding and effect compartment mechanism, as both models have an additional compartment between the plasma compartment and the compartment that is linked directly to the effect. The parameter values, OFVs and AICs are given in Table 6. Parameter values and objective function values of the tested EC–TB_PL_ models describing the EEG data, based on plasma concentrations. CV denotes the coefficient of variation as percentage. OFV denotes the Objective Function Value, AIC denotes the Akaike Information Criterion. *ω*^2^ and *σ*^2^ denote the variances of the exponential IIV distribution and the proportional error distribution, respectively. Model EC–TB_PL_1 was selected as best model on basis of the AIC, but this AIC is still 39 points higher than Model EC_PL_1. The uncertainty in the parameter estimate of the *K*_*D*_ in the presence of the Pgp blocker (*K*_*D*_–*Pgp*) is rather high with 93%, but this was allowed to test the conclusion that none of the binding models (TB_PL_1–TB_PL_5 and EC–TB_PL_1 to EC–TB_PL_5) yielded lower AICs than the best effect compartment model (EC_PL_1) in a conservative manner.

**Table 6:** Parameter values and objective function values of the tested EC–TB_PL_ models describing the EEG data, based on plasma concentrations

	Parameter definition	EC–TB_PL_1 selected model	EC–TB_PL_2 no Pgp effect	EC–TB_PL_3 Pgp on k_eo_	EC–TB_PL_4 k_off_ = 1	EC–TB_PL_5 slope = 0
OFV		44,790.9	44,880.3	44,873.8	45,008.2	45,235.3
AIC		44,808.9	44,896.3	44,891.8	45,024.2	45,251.3

Parameter	Parameter definition	Value (%CV)	Value (%CV)	Value (%CV)	Value (%CV)	Value (%CV)
*k*_*off*_ (/min)	Dissociation rate constant	0.0275 (14)	0.0243 (14)	0.0247 (14)	1 FIX	0.0400 (9)
*k*_*eo*_ (/min)	Distribution rate constant to effect compartment	0.0327 (17)	0.0365 (12)	0.0389 (14)	0.0162 (28)	0.036 (13)
*k*_*eo*_–*Pgp* (/min)	Distribution to effect compartment with Pgp blocker	–	–	0.0265 (31)	–	–
*K*_*D*_ (nM)	Equilibrium constant	1520 (34)	1150 (30)	1110 (30)	2110 (50)	3150 (36)
*K*_*D*_–*Pgp* (nM)	Equilibrium constant with Pgp blocker	296 (93)	–	–	385 (78)	594 (47)
*E*_0_ (µV)	Baseline EEG amplitude	45.0 (4)	45.7 (4)	45.6 (4)	45.2 (4)	41.9 (4)
*E*_*max*_ (µV)	Maximal increase in EEG amplitude	31.8 (11)	30.7 (11)	30.5 (11)	34.4 (18)	37.3 (12)
*R*_*tot*_ (nM)	Target concentration	1 FIX	1 FIX	1 FIX	1 FIX	1 FIX
*slope* (µV/min)	Increase in baseline EEG over time	− 0.0276 (15)	− 0.0296 (13)	− 0.0296 (13)	− 0.0273 (14)	0 FIX
*ω*^2^ *E*_0_ (µV)	IIV variance on *E*_0_	0.111 (20)	0.116 (20)	0.116 (20)	0.111 (19)	0.129 (18)
*σ* ^2^ *prop*	Variance of proportional error	0.057 (7)	0.0565 (7)	0.0565 (7)	0.0576 (7)	0.0597 (7)

#### EC_ECF_, TB_ECF_, IE_ECF_ and DE_ECF_ model fitting

**Fig. 4:**
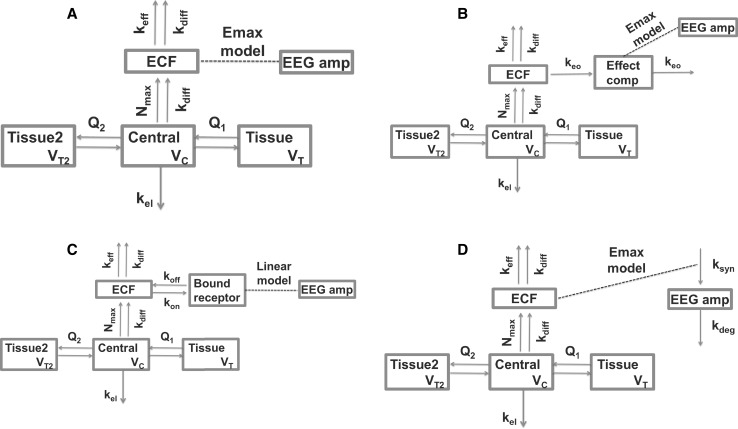
Schematic representation of the EC_ECF_, TB_ECF_, IE_ECF_ and DE_ECF_ model structures that were used to describe the EEG data, based on brain ECF concentrations. The different structures represent **a** the DE_ECF_ model, **b** the EC_ECF_ model, **c** the TB_ECF_ model and **d** the IE_ECF_ model, with *k*_*syn*_ being the zero-order effect generation rate constant, and *k*_*deg*_ being the first-order effect degradation rate constant. The distribution from plasma to the tissue compartments and the brain ECF compartment is described in Supplement S1. The arrows indicate morphine flows, the dotted line indicates a direct relationship

The last models that were fitted to the EEG data were based on the ECF concentrations instead of the plasma concentrations. Various model structures were tested, as shown in Fig. 4. To compare the model fits based on ECF concentrations (EC_ECF_1, TB_ECF_1, IE_ECF_1 and DE_ECF_1) with the model fits that were based on plasma concentrations (EC_PL_, TB_PL_ and EC–TB_PL_), the best plasma model (EC_PL_1) was fitted to the limited dataset that included only animals with ECF data. This model fit was compared to the ECF-based model fits on basis of their AICs, as shown in Table 7.

**Table 7:** Parameter values and objective function values of the tested models describing the EEG data, based on ECF concentrations

	Parameter definition	EC_PL_1 ref. model	TB_ECF_1 binding model	DE_ECF_1 direct effect	EC_ECF_1 effect compartment	IE_ECF_1 indirect effect
OFV		25,996.1	26,284.1	26,284.0	26,255.1	26,240.3
AIC		26,118.1	26,300.1	26,300.0	26,273.1	26,258.3

Parameter	Parameter definition	Value (%CV)	Value (%CV)	Value (%CV)	Value (%CV)	Value (%CV)
*k*_1*e*_ (/min)	Distribution to transit compartment	0.0457 (35)	–	–	–	–
*k*_*eo*_ (/min)	Distribution from effect compartment	0.0377 (41)	–	–	0.161 (40)	–
*k*_1*e*_–*Pgp* (/min)	Distribution to transit with Pgp blocker	0.0647 (36)	–	–	–	–
*k*_*eo*_–*Pgp* (/min)	Distribution from effect with Pgp blocker	0.0155 (77)	–	–	–	–
*E*_0_ (µV)	Baseline EEG amplitude	47.6 (6)	48.9 (6)	48.9 (6)	49.1 (6)	49.1 (6)
*E*_*max*_ (µV)	Maximal increase in EEG amplitude	27.5 (20)	32.7 (17)	23.4 (18)	24.9 (19)	25.4^a^ (36)
*E*_*max*_–*Pgp* (µV)	Maximal increase in EEG amplitude with Pgp blocker	–		41.6 (14)	43.2 (15)	43.3 (42)
*EC*_50_ (nM)	concentration giving half-maximal increase in EEG amplitude	1100 (87)	–	173 (22)	182 (26)	182 (25)
*N*_*H*_	Hill factor	2.05 (49)		2.3 (41)	2.02 (43)	2.07 (43)
*slope* (µV/min)	Increase in baseline EEG amplitude over time	− 0.0235 (34)	− 0.0400 (17)	− 0.0359 (17)	− 0.0373 (19)	− 0.0377 (19)
*k*_*off*_ (/min)	Dissociation rate constant	–	0.0932 (37)	–	–	–
*K*_*D*_	Equilibrium constant	–	283 (40)	–	–	–
*K*_*D*_–*Pgp*	Equilibrium constant with Pgp blocker	–	55.9 (15)	–	–	–
*k*_*deg*_ (/min)	Degradation rate constant					0.124 (34)
*ω*^2^ *E*_0_ (µV)	Variance of IIV on *E*_0_	0.0668	0.0668 (26)	0.072 (25)	0.0696 (26)	0.0961 (26)
*σ* ^2^ *prop*	Variance of proportional error	0.0550 (10)	0.0598 (10)	0.0598 (10)	0.0593 (10)	0.059 (10)

^a^This value was estimated as the maximal *k*_*syn*_ minus baseline *k*_*syn*_ (calculated from *E*_0_ and *k*_*deg*_) and calculated by dividing the estimated value by the *k*_*deg*_

Of all the models that are described above, model EC_PL_1 has the lowest AIC. To evaluate its performance in more detail, the most relevant diagnostic plots are given in Figures S6–S10. These diagnostic plots indicate that the main trend of the data is captured, although the obtained fit is not optimal (which is especially clear from Figure S10). The small difference in AIC between the best combined EC–TB model (EC–TB_PL_1) and the best EC model (EC_PL_1) is also reflected by very similar VPC results, as shown in Figure S11. Moreover, the best model with only binding from plasma (TB_PL_4) also provided a similar VPC result (see Figure S12).

### Dose-dependency of Tmax_TO_ in a TB_PL_ model

**Fig. 5:**
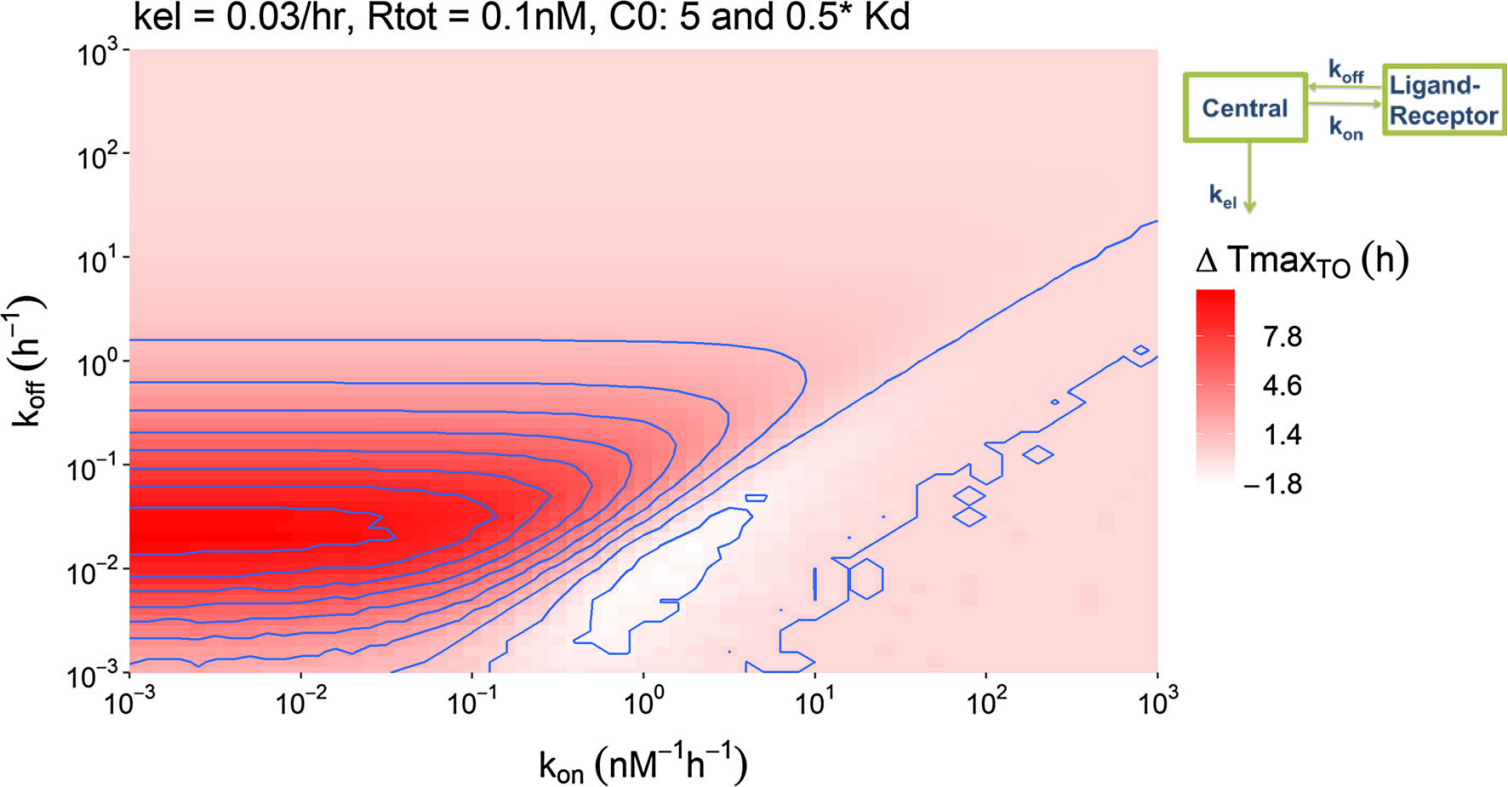
Overview of the shift in Tmax_TO_ that was observed in the simulations with the TB_PL_ model (see upper-right corner), as a result of the change in the affinity-normalized dose (leading to an initial concentration of 5 and 0.5 times the *K*_*D*_). Each pixel represents a single simulation in which the *k*_*off*_ and *k*_*on*_ value correspond to the position on the y-axis and the x-axis, respectively, and the color represents the observed shift in Tmax_TO_ in that simulation. The elimination rate constant *k*_*el*_ was 0.03/hr and the target concentration was 0.1 nM for all simulations in this figure

**Fig. 6:**
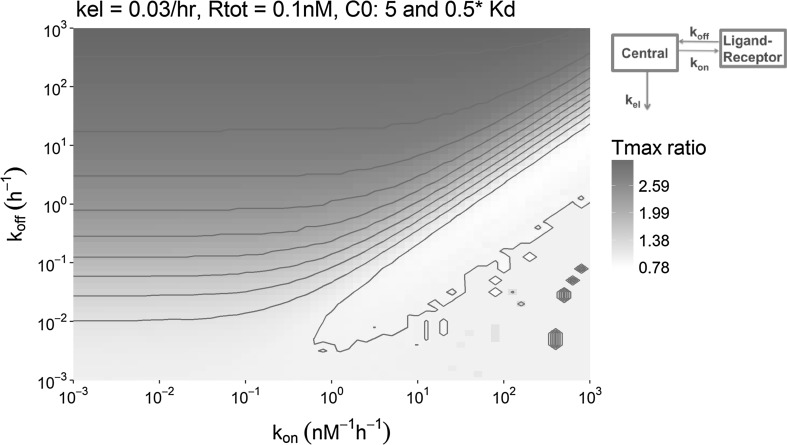
Overview of the ratio of Tmax_TO_ values that was observed in the simulations with the TB_PL_ model (see inset) as a result of the change in the affinity-normalized dose (leading to an initial concentration of 5 and 0.5 times the *K*_*D*_). Each pixel represents a single simulation in which the *k*_*off*_ and *k*_*on*_ value correspond to the position on the y-axis and the x-axis, respectively, and the color represents the observed shift in Tmax_TO_ in that simulation. The elimination rate constant *k*_*el*_ was 0.03/hr and the target concentration was 0.1 nM for all simulations in this figure

Simulations of drug-target binding in a TB_PL_ model for the range of the most relevant binding kinetics demonstrated that the observable influence of dose on Tmax_TO_, which discriminates the TB model from the EC model, is limited to a confined range of *k*_*on*_ and *k*_*off*_ combinations. As visualized in Fig. 5, if the *k*_*off*_ has a value around the elimination rate constant of 0.03/h, ∆Tmax_TO_ is maximal. Also, the initial drug concentration *C0* should not be above a specific threshold value which is approximately equal to the target concentration. The absolute ∆Tmax_TO_ for different doses (as shown in Fig. 5) will be most relevant for the identification of the dose-dependent ∆Tmax_TO_ in a PKPD modelling study. However, for the understanding of the underlying determinants of this shift in ∆Tmax_TO_, the ratio of the ∆Tmax_TO_ values belonging to the two doses should also be considered, as shown in Fig. 6. For example, if the two different Tmax_TO_ values obtained from the two doses are 1 and 3 min, their ratio is 3, but the absolute difference is 2 min. If the two Tmax_TO_ values are 1 and 3 h, their ratio is still 3, but the difference is now 2 h. In this latter case, the influence of the dose on the Tmax_TO_ will be more easily identified. Representative example simulations that can help to understand the characteristics of Fig. 5 are provided in Supplement S2.

**Fig. 7:**
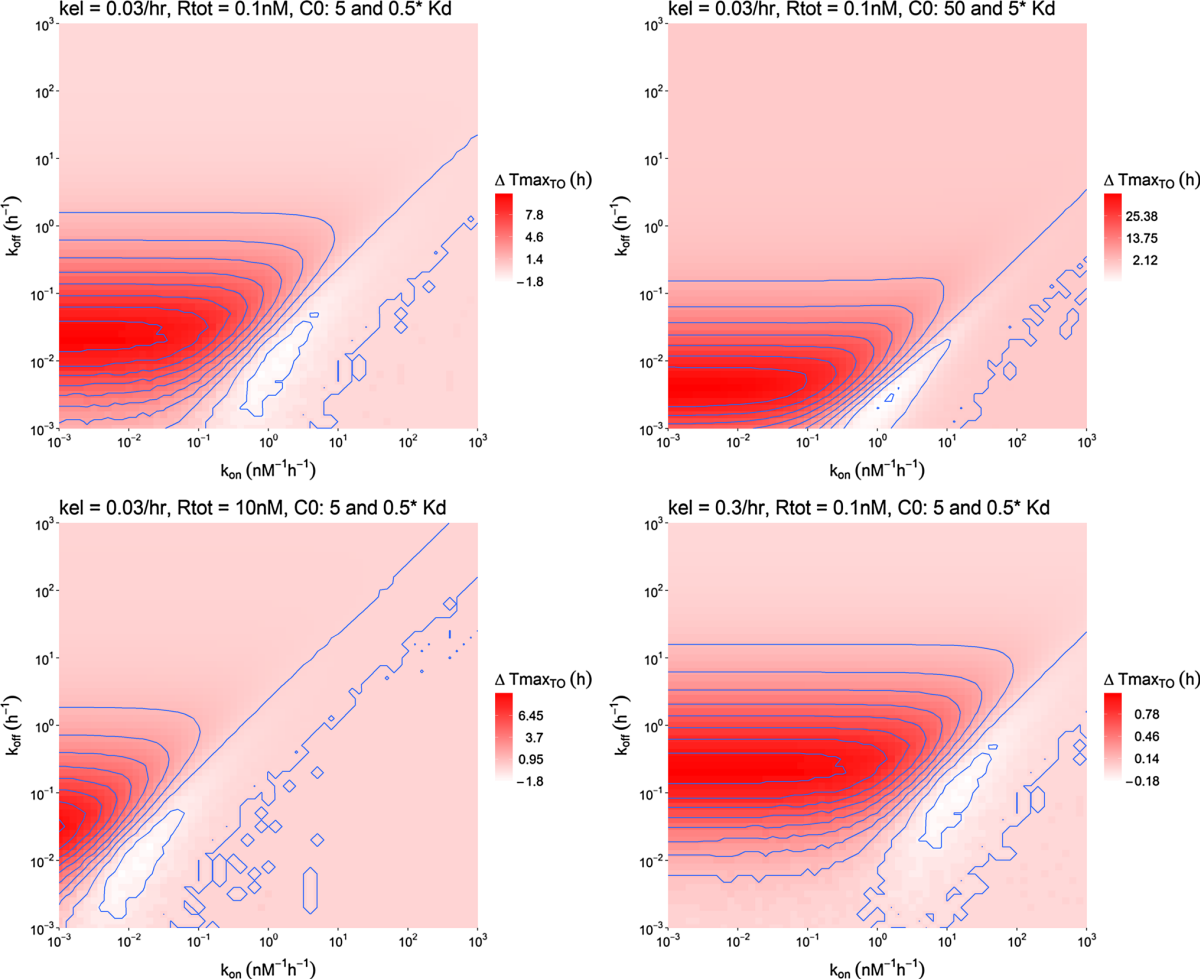
Overview of the ∆Tmax_TO_ that was observed in the simulations as a result of the change in the affinity-normalized dose for different combinations of parameter values as indicated above the panels. Each pixel represents a single simulation in which the *k*_*off*_ and *k*_*on*_ value correspond to the position on the y-axis and the x-axis, respectively, and the color represents the observed shift in Tmax_TO_ in that simulation. All panels vary only one other parameter compared to the upper left panel

Interestingly, the relationship between the ∆Tmax_TO_, the elimination rate constant, the target concentration and the dose could be approximated mathematically for the upper region, the lower-left region and the lower-right region of Fig. 5 as presented in Supplement S3. From this analysis, it follows that for the upper half of Fig. 5, where the *k*_*off*_ is much larger than the *k*_*el*_, Tmax_TO_ is always small, and a significant ∆Tmax_TO_ will thus not be observed. For the lower and the lower-right part of Fig. 5, where the *k*_*off*_ is much smaller than the *k*_*el*_, it is found that Tmax_TO_ does not depend on the dose. More specifically, when the initial drug concentration is much lower than the target concentration (and *k*_*off*_ is smaller than *k*_*el*_), the Tmax_TO_ is merely determined by the *k*_*el*_. On the other hand, when the initial drug concentration is much larger than the target concentration (and *k*_*off*_ is smaller than *k*_*el*_), the Tmax_TO_ is given by a relation between *k*_*off*_ and *k*_*el*_. This relationship between the ∆Tmax_TO_, the elimination rate constant, the target concentration and the dose is illustrated in Fig. 7.

## Discussion

In this study, TB and EC models were compared to describe the delay between morphine plasma concentrations and EEG effects for three different dose levels. Model discrimination was difficult to obtain and selection of the best model (the EC_PL_ model in this study) was only possible on basis of the objective function value differences. Moreover, simulations with the TB_PL_ model showed that a shift in Tmax_TO_ with increasing doses, the distinctive future of the TB model compared to the EC model, only occurs for a limited range in parameter values. Both a *k*_*off*_ value much smaller and much larger than the *k*_*el*_ value and a target concentration larger than the initial drug concentration decrease this shift in Tmax_TO_ towards zero.

Since the simulations show that the Tmax_TO_ does not depend on the dose for *k*_*off*_ values much lower than the *k*_*el*_ and target concentrations much higher than the initial drug concentration, this means that the TB_PL_ model for these parameter values behaves like an EC_PL_ model, with a first order increase and decrease in the concentration that is linked to the effect. Together with the small differences in EC and TB model fits to the morphine EEG data, this shows that for many parameter combinations, a TB model gives rise to similar drug effect profiles as an EC model. This means that neither a successful fit of a TB or EC model necessarily supports the relevance of target binding or target site distribution, respectively, while a single successful fit is often presented as such support [11, 23, 39]. To obtain support for one of the two mechanisms, both models should be fitted to the data and compared on basis of objective metrics such as the AIC. This approach demonstrated the added value of the combined EC–TB_PL_ model compared to the EC_PL_ and the TB_PL_ model for buprenorphine and AR-HO47108 [13, 29]. However, this method also demonstrated that the TB_PL_ model performed similarly as the EC_PL_ model for eight calcium antagonists [14] and that the EC model performed similarly as the EC–TB_PL_ model for fentanyl [13]. This demonstrates that even if objective metrics are used, discrimination between two models is not always possible. Moreover, obtained model discrimination strictly informs on the data fit of each model, not directly on the plausibility of the represented mechanism. This lack of discrimination between TB and EC models means that a visually satisfying model fit of the EC model does not indicate that the TB will be not applicable. The TB model should therefore be considered and tested more often as mechanistic alternative to the EC model to find the best fitting model. Moreover, the TB model parameters can be measured partially in vitro/ex vivo, which enables a better in vitro–in vivo extrapolation (IVIVE).

In this study, the models based on brain ECF concentrations did not perform better than the models based on plasma concentrations. One would expect that the brain ECF concentrations would reflect the target site concentration better than the plasma concentrations, especially if brain distribution is relatively slow and nonlinear, as it was in this study. The inferior performance of the brain ECF-based models might be explained by the extremely high variability in the brain ECF data of the 4 mg/kg dose group, as shown in Figure S5. However, a direct effect model (DE_ECF_1) could be identified from the brain ECF concentrations and showed an only 39 points higher AIC than the best model IE_ECF_1, while an IE model fit could not be obtained from the plasma concentrations, indicating that the ECF concentrations reflect the target site concentration more closely compared to the plasma concentrations. This is in line with the relevance of drug concentrations in the brain for CNS effects that has been demonstrated by several other studies [40–43] and the difference between plasma and brain concentrations that has been identified for several compounds [44]. In all our target binding models, a linear target occupancy–effect relationship had to be assumed to keep the model parameters identifiable. Such a linear relationship has been observed and can be expected unless for full agonists in tissues with relatively high target concentrations compared to the concentration of signal transduction molecules (i.e. for a high receptor reserve) [24].

Only a one compartment pharmacokinetic model was used in this study in combination with the simplest TB_PL_ model to investigate the ∆Tmax_TO_. The same principles are expected to apply if the TB_PL_ model has a two-compartment or three-compartment pharmacokinetic model or with target turnover and signal transduction models, but the parameter range for which Tmax_TO_ shifts with a change in dose might be different compared to the model used in the simulations. In analogy to Fig. 7, for the combined EC–TB model one would expect that to obtain a significant ∆Tmax_TO_ and to identify the TB model in addition to the EC model, the *k*_*e*0_ should be in the same order of magnitude as the *k*_*off*_ if the maximal drug concentration is around or below the *K*_*D*_. This is indeed the case for the two successful examples of an EC–TB_PL_ fit: for buprenorphine, the *k*_*e*0_ was 0.0242/min and the *k*_*off*_ was 0.0731/min [13] and for AR-HO47108, the *k*_*e*0_ was 0.0351 for the drug and 0.00749 for its metabolite and the *k*_*off*_ was 0.00303 and 0.00827/min, respectively [29]. On the other hand, the combined EC–TB model EC–TB_PL_1 that was identified in this study for morphine also showed a similar value for *k*_*e*0_ and *k*_*off*_ (0.0327 and 0.275, respectively), but this model was not better than the EC model EC_PL_1. In comparison with our one compartment pharmacokinetics model with intravenous dosing, especially the absorption or the distribution phase into the target site could pose additional limiting factors that prevent a shift in Tmax_TO_ with increasing doses.

One of the most important advantages of the EC model is that it only requires one parameter, *k*_*e*0_. However, the EC model most often needs to be combined with an Emax model, which also requires two or three parameters, *Emax*, *EC*_50_ and possibly the hill factor. The binding model has three parameters, *k*_*on*_, *k*_*off*_ and *R*_*tot*_, and needs at least 1 additional parameter, *Emax*, to convert occupancy predictions to effect predictions. One or two additional parameters might be required to describe a nonlinear target occupancy-effect relationship, which is required in case of a high efficacy and receptor reserve [24]. The discrimination between the two nonlinearities in such cases might be hard or impossible to obtain. However, *k*_*on*_ and *k*_*off*_ can be obtained from in vitro experiments and *R*_*tot*_ from ex vivo experiments. Especially the identification of *R*_*tot*_ from ex vivo data can help to reduce the difficulties with parameter identifiability as often associated with the TB model [45]. It should be noted that the measurement of in vitro target binding only helps to explain/describe the delay between pharmacokinetics and pharmacodynamics if the target binding kinetics are the rate-limiting step in this delay in vivo. As biologics often display lower dissociation rate-constants and their target binding is more often affecting the pharmacokinetics compared to small molecules, as exemplified by the multitude of TMDD model applications in this area, one could expect that the TB model is mostly relevant for biologics. However, small molecules can also have low dissociation rate-constants, as exemplified by the irreversible binders aspirin and omeprazole and the target association-dissociation of the semi-synthetic opioid buprenorphine. Moreover, the increasing interest in the pharmaceutical industry to design small molecules with low dissociation rate-constants can lead to an increase in the number of such molecules in drug discovery and development and the associated modeling efforts.

In summary, the limited difference between TB and EC models should be taken into account in the evaluation of historical and the design of new modelling studies. By informing the TB models with in vitro data, TB models can help to translate between in vitro and in vivo studies if the target binding is a rate-limiting step. The combination of parameter values for which the Tmax_TO_ in the target binding model is dependent on the dose is limited to *k*_*off*_ values around the elimination rate constant and to target concentrations lower than the initial drug concentration. Although the combination of multi-compartment pharmacokinetics models, TB models and target turnover models might affect the parameter range were the Tmax_TO_ is dependent on the dose, this study is a first indication that such limitations should be taken into account for understanding TB models.

## Conclusion

In this study, it was shown that successful fitting of a TB or EC model is not enough support to assume the relevance of target binding or target site distribution. Moreover, for a one-compartment pharmacokinetic model with target binding, the ∆Tmax_TO_ for changing doses can only be identified if the *k*_*off*_ has a value around the pharmacokinetic elimination rate constant and the target concentration is lower than the initial drug concentration. The Tmax_TO_ is determined by the rate of target binding relative to the decline rate of unbound drug and unbound target concentrations. These findings indicate that the relatively sparse occurrence of target binding models in literature does not discredit the relevance of target binding kinetics. This study also shows that a TB and EC model might be similar for the tested dose range and pharmacokinetic conditions, while extrapolation to different conditions might result in different effect versus time profiles for the TB and EC model. In conclusion, the identification of the appropriate model is important and target binding models should be tested more often to increase the translation between in vitro and in vivo studies and to increase the predictive power of developed PKPD models.

## Electronic supplementary material

Below is the link to the electronic supplementary material.

Supplementary material 1 (DOCX 2773 kb)

## Abbreviations

AIC: Akaike information criterion
CNS: Central nervous system
CV: Coefficient of variation
DE: Direct effect
ECF: Extracellular fluid
EC: Effect compartment
IE: Indirect effect
GOF: Goodness of fit
IIV: Inter-individual variability
OFV: Objective function value
Pgp: P-glycoprotein
PKPD: Pharmacokinetic–pharmacodynamic
TB: Target binding
Tmax_PD_: Time between dosing and maximal drug effect
Tmax_TO_: Time between dosing and maximal target occupancy
∆Tmax_TO_: Tmax_TO_ of the lower dose − Tmax_TO_ of the higher dose
VPC: Visual predictive check
