# Correction to: Modelling the delay between pharmacokinetics and EEG effects of morphine in rats: binding kinetic versus effect compartment models

**DOI:** 10.1007/s10928-018-9604-y

**Published:** 2018-08-31

**Authors:** Wilhelmus E. A. de Witte, Vivi Rottschäfer, Meindert Danhof, Piet H. van der Graaf, Lambertus A. Peletier, Elizabeth C. M. de Lange

**Affiliations:** 1 Division of Pharmacology, Leiden Academic Centre for Drug Research, Leiden University, 2333 CC Leiden, The Netherlands; 2 Mathematical Institute, Leiden University, 2333 CA Leiden, The Netherlands; 3 Certara Quantitative Systems Pharmacology, Canterbury Innovation Centre, Canterbury, CT2 7FG UK

## Correction to: Journal of Pharmacokinetics and Pharmacodynamics (2018) 45:621–635 10.1007/s10928-018-9593-x

The original version of this article was published open access. Unfortunately, due to a technical issue, the copyright holder name in the online version (HTML and XML) is incorrectly published as “Springer Science+Business Media, LLC, part of Springer Nature 2018”. Instead, it should be “The Author(s) 2018”.

The original article has been corrected.

