# Supplementary material for: Modelling the delay between pharmacokinetics and EEG effects of morphine in rats: binding kinetic versus effect compartment models
Source: J Pharmacokinet Pharmacodyn. 2018 May 18;45(4):621–35. doi: 10.1007/s10928-018-9593-x (PMC6061075; doi:10.1007/s10928-018-9593-x)
Supplement: Supplementary file 1 — Supplementary material 1 (DOCX 2773 kb) [file 10928_2018_9593_MOESM1_ESM.docx]

## Supplement S 1. Morphine pharmacokinetics and pharmacodynamics model fits, GOF plots and VPCs

## Plasma concentration modelling

A three-compartment model (Figure S 1) was identified as the best model with respect to the AIC and the individual fits. The goodness of fit of this model is illustrated in Figure S 2 and Figure S 3.


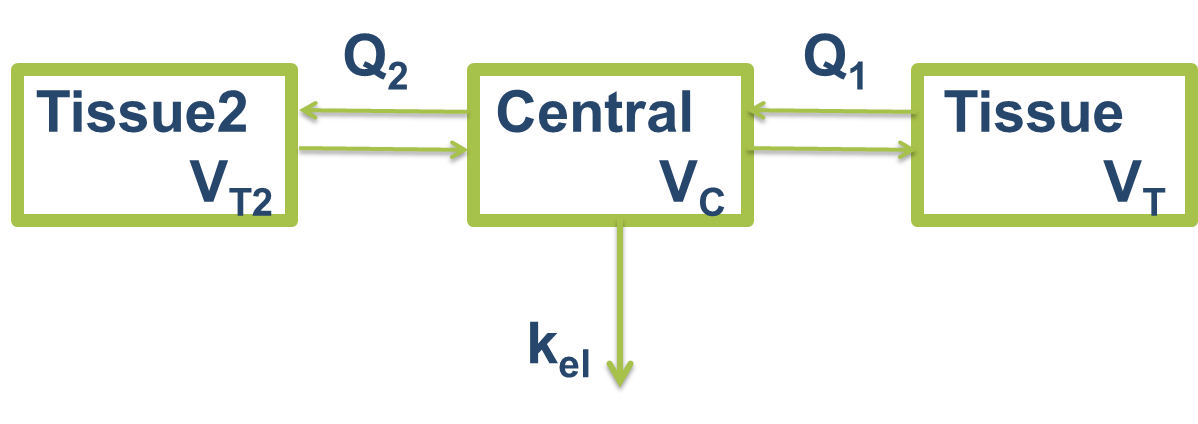


Figure S 1. Schematic representation of the three-compartment model structure that was used to describe the morphine plasma concentrations over time.

The differential equations of the model in Figure S 1 are given in equations 1-3. In these equations, *A_c_*, *A_2_* and *A_3_* represent the amount of drug in the central, second and third compartment, respectively. k_el_ represents the first order rate constants of elimination Q1 and Q2 represent the distribution clearances between the compartments. The relation between the parameters in equations 1-3 and the estimated parameters as given in Table S 1 is shown in equation 4-8. V_c_, V_2_ and V_3_ represent the volumes of the respective compartments and CL, Q12 and Q13 represent the clearances of elimination and distribution between compartments. The amounts in all compartments are 0 at time t = 0.

$\frac{dA_{c}}{dt}= - k_{el} \cdot A_{c}- k_{in} \cdot A_{c}- k_{in2} \cdot A_{c}+ k_{out} \cdot A_{2}+ k_{out2} \cdot A_{3}$

$\frac{dA_{2}}{dt}= k_{in} \cdot A_{c}- k_{out} \cdot A_{2}$

$\frac{dA_{3}}{dt}= k_{in2} \cdot A_{c}- k_{out2} \cdot A_{3}$

$k_{el}= \frac{CL}{V_{c}}$

$k_{12}= \frac{Q1}{V_{c}}$

$k_{13}= \frac{Q2}{V_{c}}$

$k_{21}= \frac{Q1}{V_{2}}$

$k_{31}= \frac{Q2}{V_{3}}$

The goodness of fit of this model is illustrated in Figure S 2 and Figure S 3. Inter individual variability (IIV) was estimated for 4 of the estimated model parameters. Attempts to add IIV on more parameters resulted in a failing covariance step while the drop in OFV was limited (9 points). Comparison to the 2-compartment model with the lowest OFV value that was tested demonstrated better individual fits and a 109 points lower OFV for the 3-compartment model.


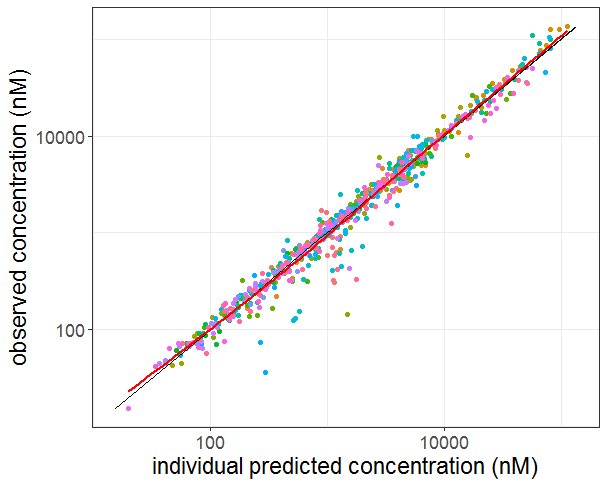

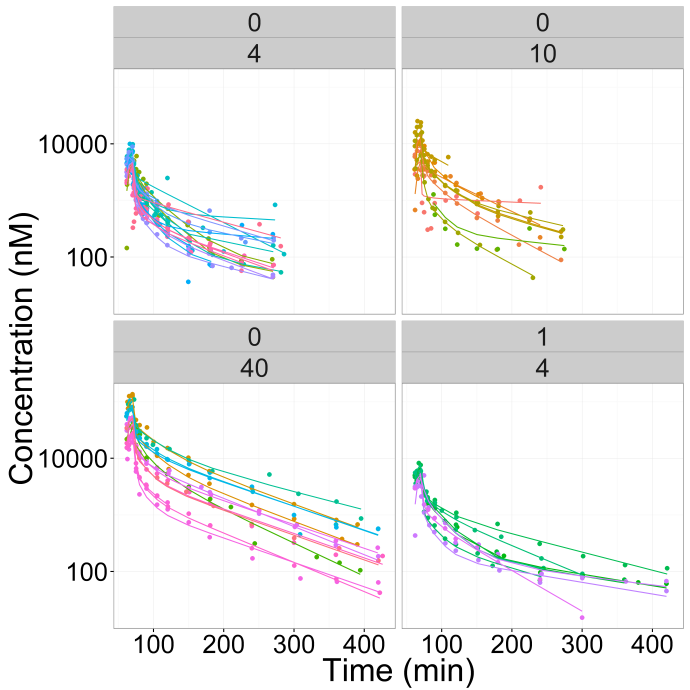


With inhibitor

4 mg/kg

No inhibitor

40 mg/kg

No inhibitor

10 mg/kg

4 mg/kg

No inhibitor

Figure S 2. Diagnostic plots of the plasma concentration fits. Left panel: Overview of observed (dots) and predicted (lines) concentrations. Upper panel labels indicate the dose in mg/kg and lower panel labels the presence or absence of Pgp inhibitor GF120918. Right panel: relation between observed and individual predicted plasma concentrations on a double logarithmic scale. The red line represents the loess smoother of the points, the black line is the line of identity. The colors are for visual distinction of the different IDs.


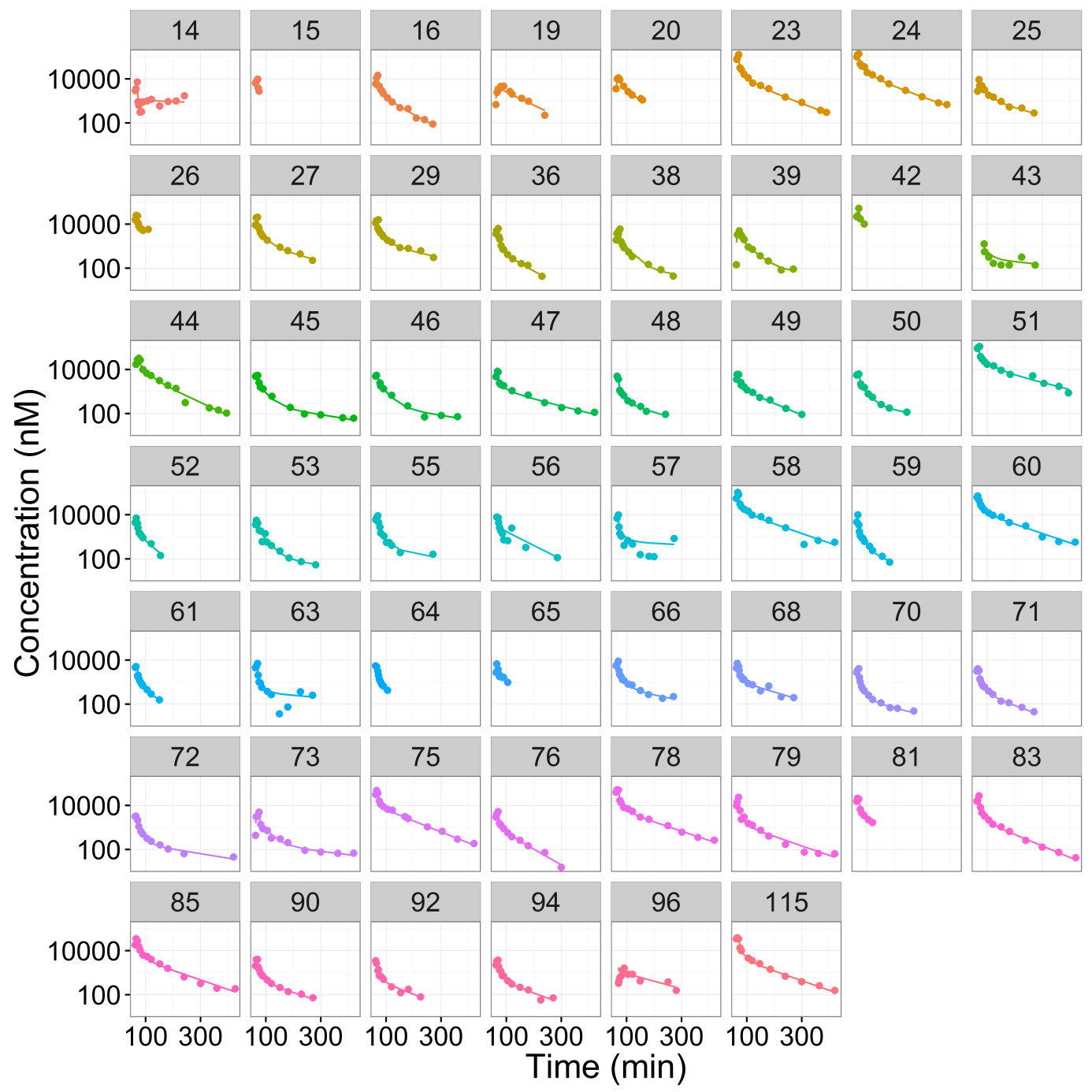


Figure S 3. Individual profiles of observed (dots) and predicted (lines) concentrations on a semi-logarithmic scale. Panel labels indicate the animal ID number. The colors correspond to the ID numbers.

Table S 1. Parameter values and objective function values of the tested models for the plasma concentrations.

|  | Parameter definition | 2-cmp model | 3-cmp model  4 IIV parameters | 3-cmp model  5 IIV parameters |  |
| --- | --- | --- | --- | --- | --- |
| OFV |  | 9314 | 9205 | 9194 |  |
| parameter |  | **Value (CV)** | **Value (CV)** | **Value (CV)** |  |
| *CL* (L/min) | Plasma Clearance | 0.0300 (8) | 0.028 (22) | 0.028 |  |
| *V_C_* (L) | Plasma volume | 0.200 (11) | 0.17 (49) | 0.12 |  |
| *Q1* (L/min) | Intercompartmental clearance | 0.0432 (10) | 0.019 (66) | 0.056 |  |
| *V2* (L) | Peripheral compartment volume | 1.15 (10) | 1.3 (48) | 0.51* |  |
| *Q2* (L/min) | Intercompartmental clearance | - | 0.031 (30) | 0.020 |  |
| *V3* (L) | peripheral compartment volume | - | 0.36 (32) | 1.4* |  |
|  |  |  |  |  |  |
| *ω^2^ CL* | IIV variance on CL | 0.34 (23) | 0.33 (25) | 0.35 |  |
| *ω^2^ V_C_* | IIV variance on V_C_ | 0.55 (27) | 0.63 (35) | 0.15 |  |
| *ω^2^ Q1* | IIV variance on Q1 | 0.28 (39) | 0.62 (33) | 0.72 |  |
| *ω^2^ V2* | IIV variance on V2 | 0.32 (31) | 0.66 (28) | 0 FIX* |  |
| *ω^2^ Q2* | IIV variance on Q2 |  |  | 0.79 |  |
| *ω^2^ V3* | IIV variance on V3 |  |  | 0.57* |  |
|  |  |  |  |  |  |
| *σ^2^ prop* | variance of proportional error | 0.0766 (15) | 0.79 (14) | 0.071 |  |
| *σ^2^ add* | variance of additive error | 1710 (28) | 0 FIX | 7.2 |  |

* To get the best model fit, *V2* and *V3* were estimated here as the ratio of *V2* and *V1* and the ratio of *V3* and *V2*, respectively. The displayed inter-individual variability parameter values were implemented as variability on the *V2/V1* and the *V3/V2* ratios. The displayed values in this table are derived from the estimated ratios.

## ECF concentration modelling

Various structural models were tested for the description of the ECF concentrations, including a two-compartent model (ECF and “deep brain”) and a target binding model (ECF-unbound and ECF bound). The best combination of OFV, parameter estimate uncertainty and diagnostic plots was obtained with the original one compartment ECF model, with passive first-order in- and outward distribution, saturable influx and first-order efflux (Figure S 4). As the parameters for the plasma concentrations were fixed, the only additional equation is given in equation 9, in which *A_ECF_* and *V_ECF_* refer to the amount and volume of the ECF compartment, respectively, *k_diff_* and *k_eff_* represent first order influx and efflux rate constants, *Nmax* represents the maximal saturable influx rate and *C_50_* is the plasma concentration at which the saturable influx is half-maximal. *A_ECF_*/*V_ECF_* is 0 at time t =0.

$\frac{d\frac{A_{ECF}}{V_{ECF}}}{dt}= k_{diff} \cdot\left( \frac{A_{1}}{V_{1}}-\frac{A_{ECF}}{V_{ECF}} \right)+\frac{N_{max} \cdot\frac{A_{1}}{V_{1}}}{C_{50}+ \frac{A_{1}}{V_{1}}}- k_{eff} \cdot\frac{A_{ECF}}{V_{ECF}}$

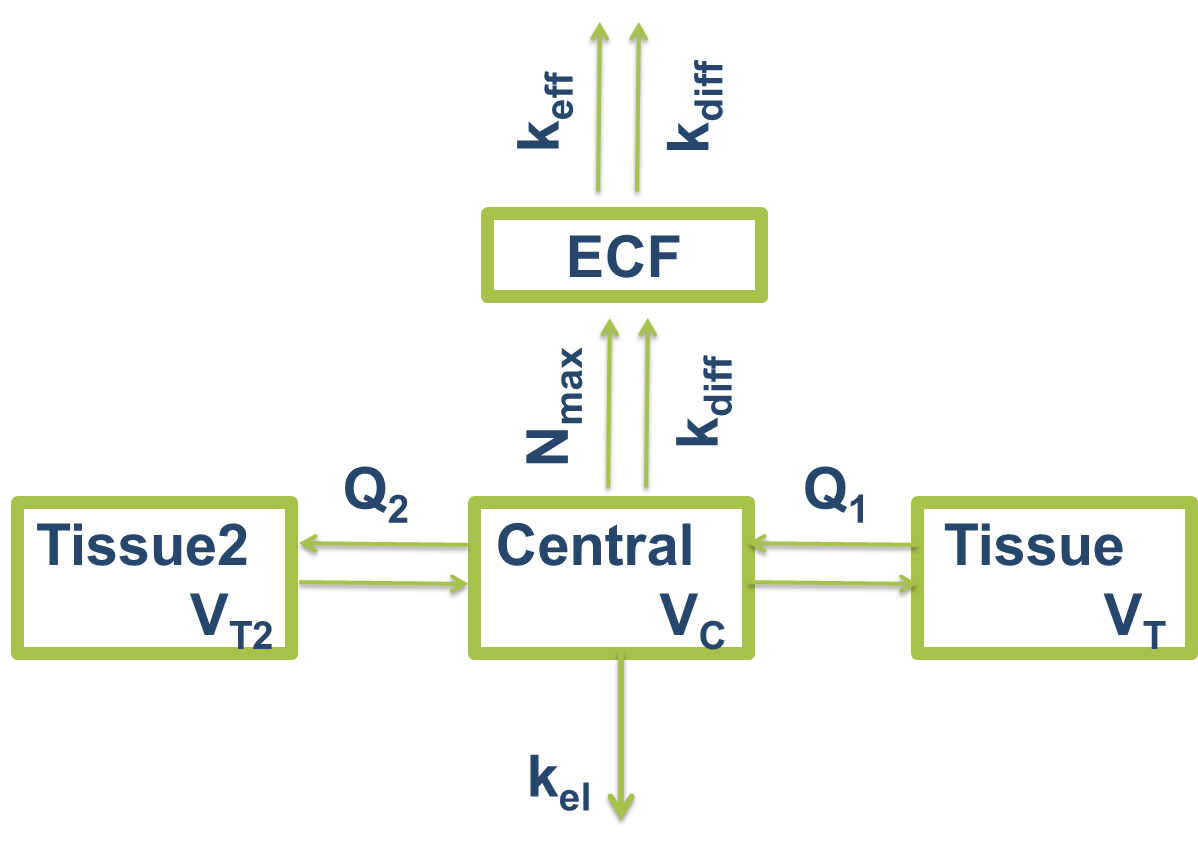


Figure S 4. Schematic representation of the model structure that was used to describe the morphine ECF concentrations over time. *k_diff_* = first-order in- and outward distribution rate constant. *k_eff_* first-order efflux rate constant. *Nmax* = zero-order maximal saturable influx rate constant.

Different versions of this model were tested in which the inter-individual variability was tested on different parameters and the influence of Pgp was estimated. Estimating the influence of Pgp did not reduce the OFV enough, so the final model did not include the influence of Pgp and had IIV estimated for k_diff_ and N_max_. The diagnostic plots for the evaluation of the fit of this model is given in Figure S 5.

Table S 2. Parameter values and objective function values of the tested models for the ECF concentrations. CV denotes the coefficient of variation as percentage. OFV denotes the Objective Function Value, *k_diff_* and *k_eff_* denote first-order distribution rate constants, *Nmax* denotes the maximal non-linear distribution into the ECF compartment, *-Pgp* denotes the parameter in the presence of a Pgp inhibitor. *ω^2^* and *σ^2^* denote the variances of the exponential IIV distribution and the error distribution, respectively.

|  | Parameter definition | IIV on k_diff_, N_max_ | IIV on k_diff_, k_eff_ | IIV on k_diff_, k_eff_  Pgp on N_max_ | IIV on k_diff_, N_max_  Pgp on N_max_ |
| --- | --- | --- | --- | --- | --- |
| OFV |  | -1126 | -1096 | -1104 | -1128 |
| parameter |  | **Value (CV)** | **Value (CV)** | **Value (CV)** | **Value (CV)** |
| *k_diff_*(/min) | Diffusion rate constant | 0.0025 (17) | 0.0027 (19) | 0.0027 (17) | 0.0025 (16) |
| *k_eff_*(/min) | efflux rate constant from ECF | 0.020 (11) | 0.021 (20) | 0.0213 (24) | 0.019 (12) |
| *N_max_* (nM/min) | Maximal active influx | 2.6 (21) | 3.0 (38) | 2.2 (34) | 2.2 (29) |
| *N_max_-Pgp* (nM/min) | Maximal active influx with Pgp blocker | - | - | 4.45 (52) | 3.15 (28) |
|  |  |  |  |  |  |
| *ω^2^ k_diff_* | IIV on *k_diff_* | 0.36 (39) | 0.44 (47) | 0.44 (45) | 0.35 (39) |
| *ω^2^ k_eff_* | IIV on *k_eff_* | 0 FIX | 0.35 (108) | 0.31 (71) | 0 FIX |
| *ω^2^ N_max_* | IIV on *N_max_* | 0.42 (55) | 0 FIX | 0 FIX | 0.39 (52) |
|  |  |  |  |  |  |
| *σ^2^ prop* | Variance of proportional error | 0.11 (18) | 0.11 (22) | 0.11 (22) | 0.11 (20) |


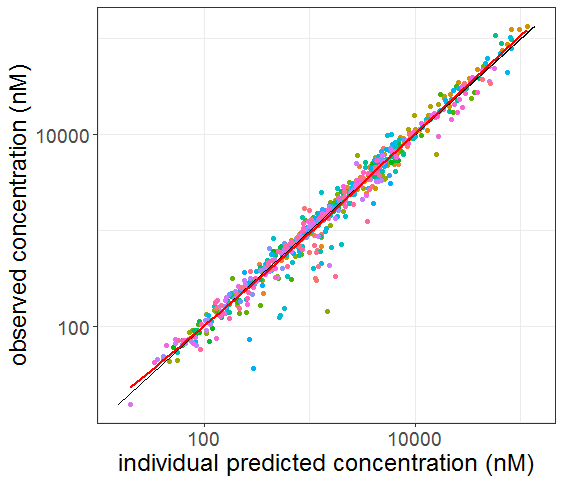

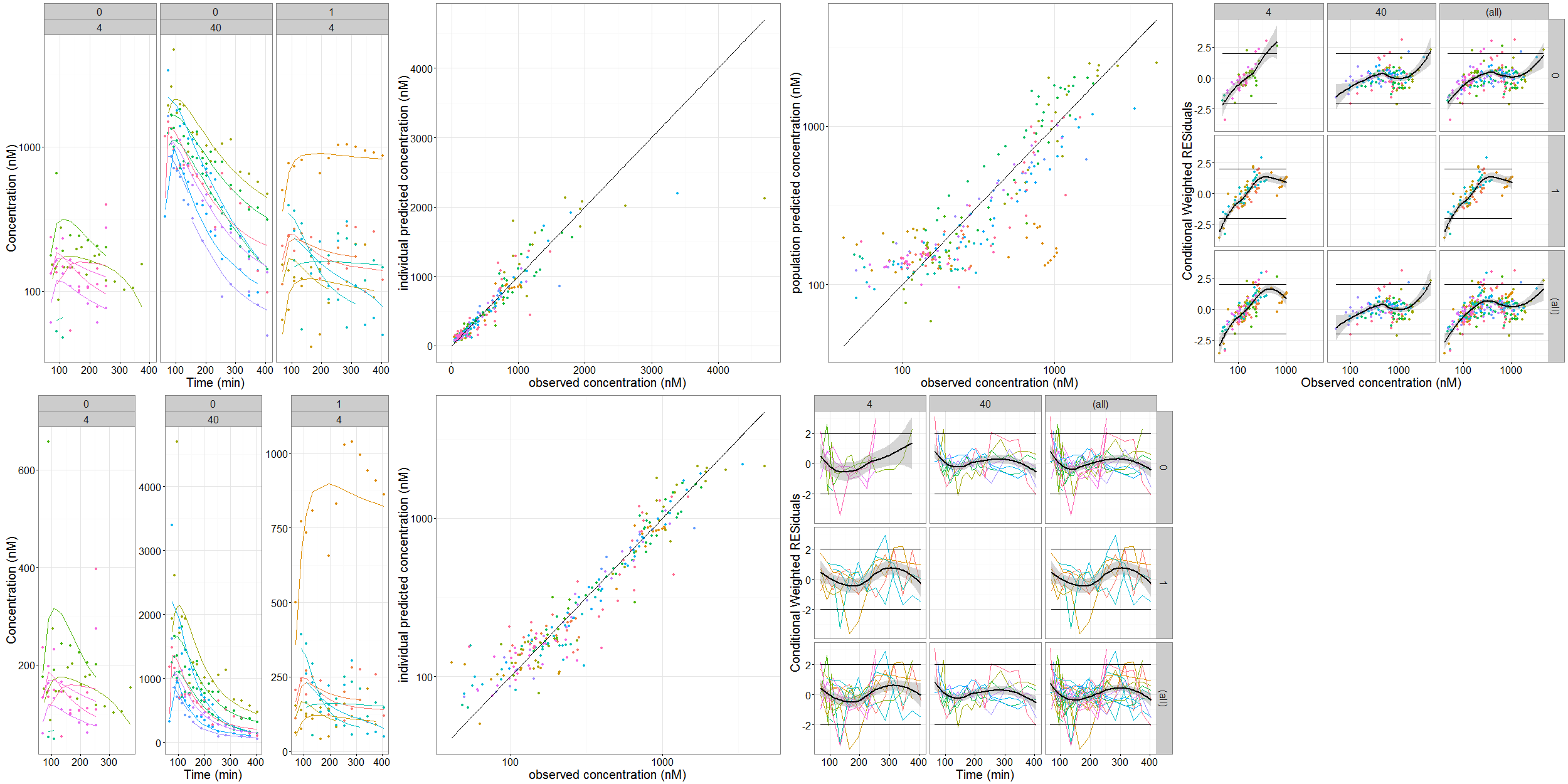


4 mg/kg

With inhibitor

40 mg/kg

No inhibitor

4 mg/kg

No inhibitor

Figure S 5. Diagnostic plots of the ECF concentration fits. Left panel: Overview of observed (dots) and predicted (lines) concentrations. lower panel labels indicate the dose in mg/kg and upper panel labels the presence or absence of Pgp inhibitor GF120918. Right panel: relation between observed and individual predicted plasma concentrations on a double logarithmic scale. The red line represents the loess smoother of the points, the black line is the line of identity. The colors are for visual distinction of the animal IDs.

## EEG effect modelling

**Model equations, Goodness of fit and VPC for model EC_PL_1**

The model equations for the connection between plasma concentrations and EEG effect are given in equations 10-12, where *A_TRANS_* and *V_TRANS_* refer to the amount of drug and the volume of the transit compartment, *A_EFF_* and *V_EFF_* refer to the amount of drug and the volume of the effect compartment *k_1e_* and *k_e0_* refer to the first order distribution rate constants into and out of the transit and effect compartment, *E0* is the baseline EEG amplitude, *slope* is the linear change of the EEG amplitude during the experiment without morphine treatment, *Emax* is the maximal increase in EEG amplitude due to morphine, *N_H_* is the hill coefficient and *EC_50_* is the morphine plasma concentration that leads to the half-maximal increase in EEG amplitude. *A_TRANS_*/*V_TRANS_* and *A_EFF_*/*V_EFF_* are 0 at time t = 0.

$\frac{d\frac{A_{TRANS}}{V_{TRANS}}}{dt}= k_{1e} \cdot\left( \frac{A_{1}}{V_{1}}-\frac{A_{TRANS}}{V_{TRANS}} \right)$

$\frac{d\frac{A_{EFF}}{V_{EFF}}}{dt}= k_{1e} \cdot\left( \frac{A_{TRANS}}{V_{TRANS}} \right)- k_{e0} \cdot\frac{A_{EFF}}{V_{EFF}}$

$Effect \left( EEG amplitude \right)= E_{0}+slope*t + \frac{E_{max} \cdot\left( \frac{A_{EFF}}{V_{EFF}} \right)^{N_{H}}}{{{EC}_{50}}^{N_{H}}+\left( \frac{A_{EFF}}{V_{EFF}} \right)^{N_{H}}}$

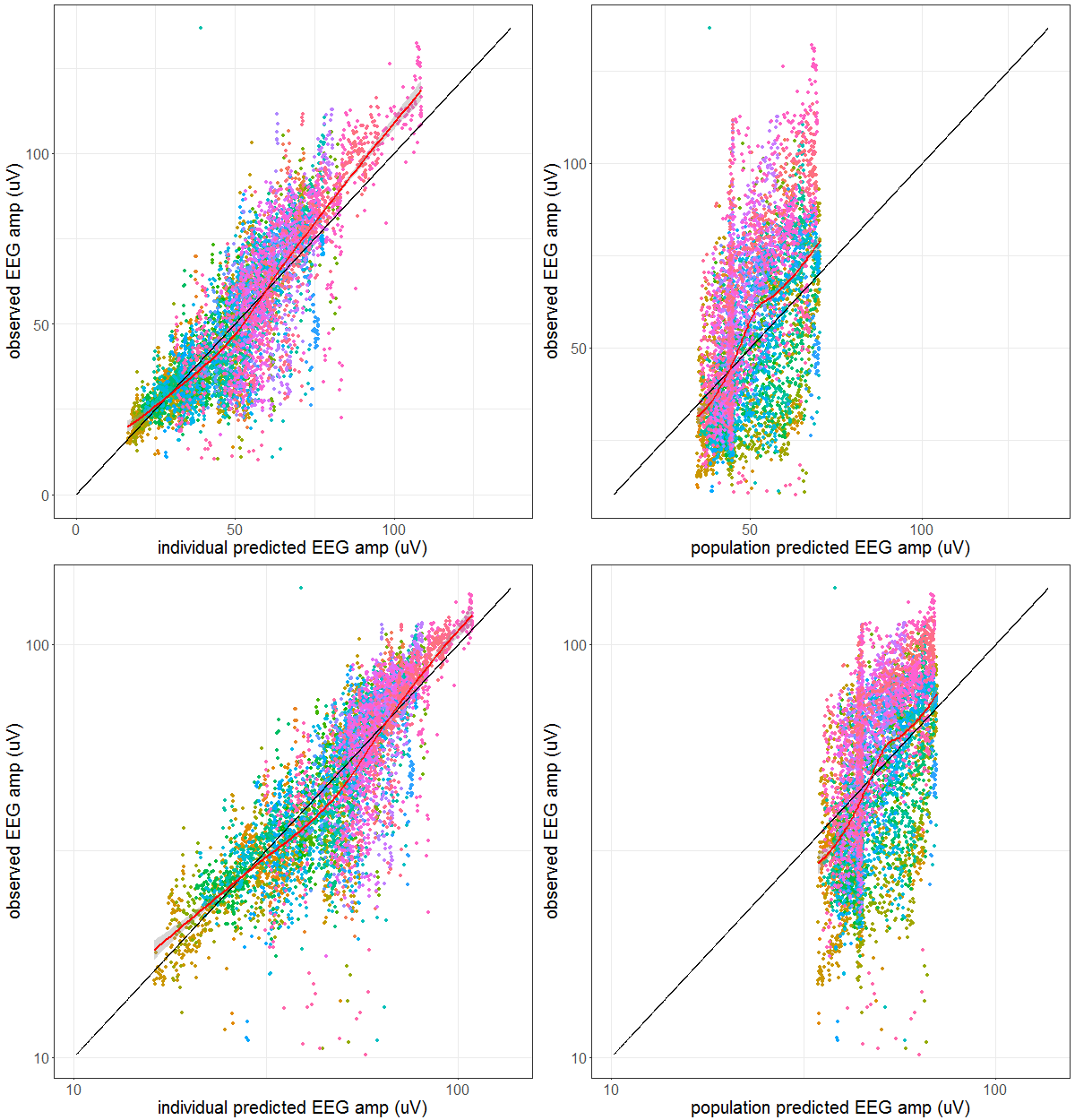


Figure S 6. Population (right panels) and individual (left panels) observed versus predicted EEG data as obtained from the model fit of model EC_PL_1. The upper panels have a linear scale and the lower panels have a logarithmic scale. The red line represents the loess smoother of the points, the black line is the line of identity. The colors are for visual distinction of the different IDs. The colors are for visual distinction of the animal IDs.


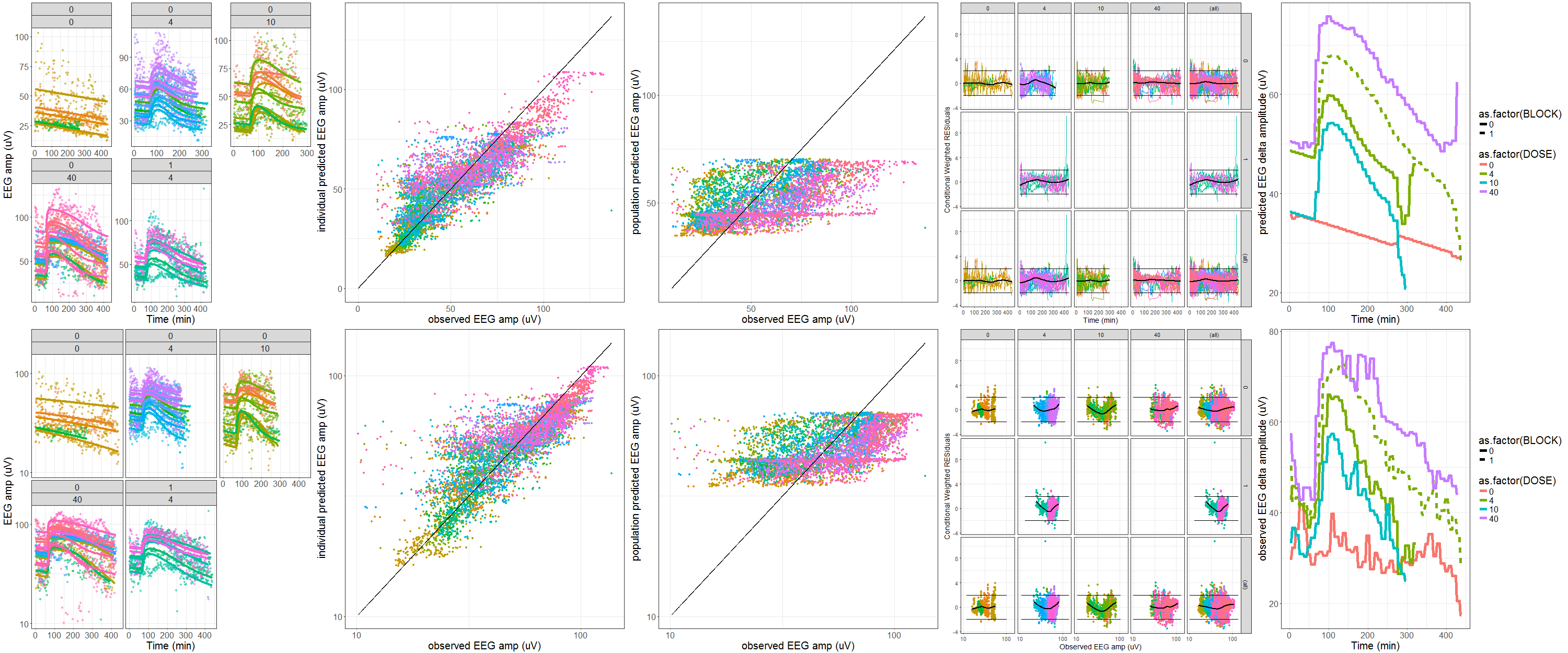


All

With inhibitor

No inhibitor

All

40 mg/kg

10 mg/kg

4 mg/kg

0 mg/kg

Figure S 7. Conditional weighted residuals versus time for the different dose groups and dose group combinations in the model fit of EC_PL_1. The top labels indicate the morphine dose in mg/kg. The side labels indicate the absence or presence of Pgp inhibitor GF120918. The columns and rows indicated with (all) display the combination of all dose groups or all Pgp inhibitor groups, respectively. The colors are for visual distinction of the animal IDs.


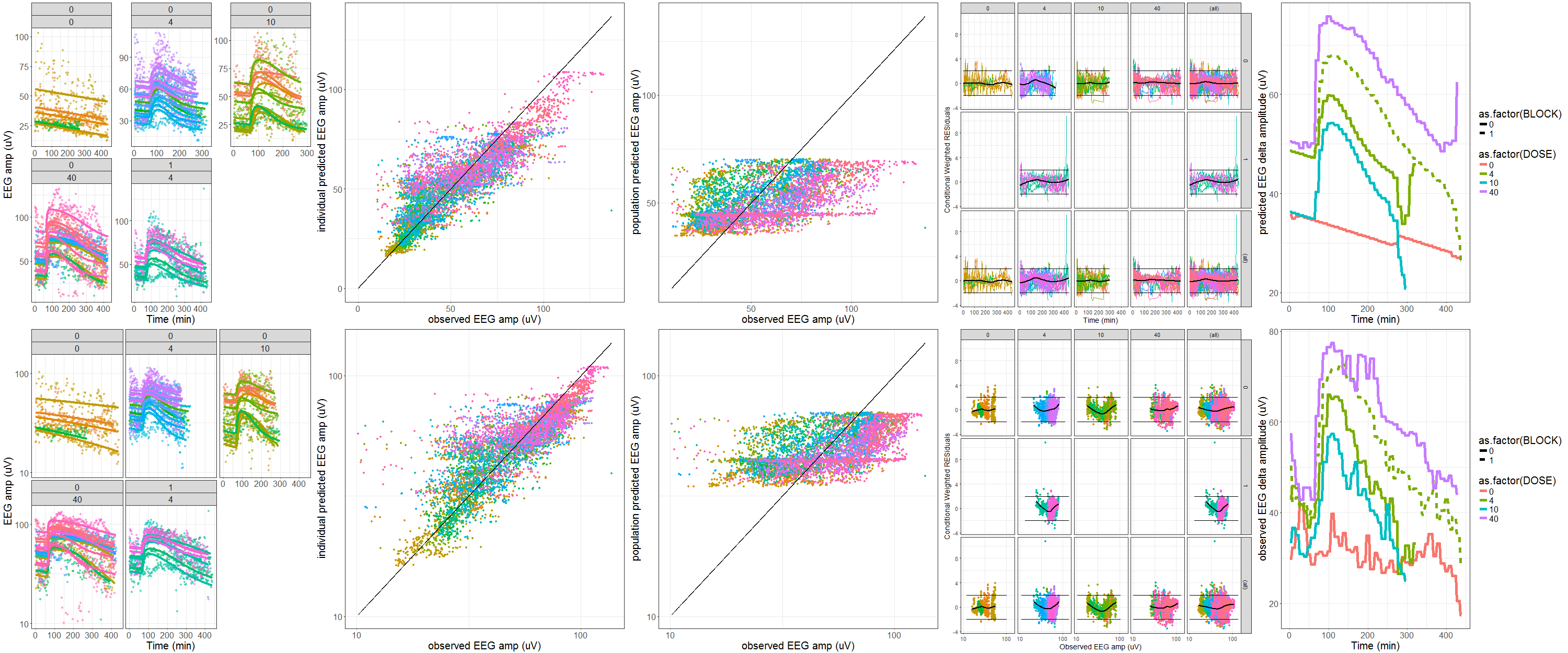


All

With inhibitor

No inhibitor

all

40 mg/kg

0 mg/kg

4 mg/kg

10 mg/kg

Figure S 8. Conditional weighted residuals versus observed EEG amplitudes. for the different dose groups and dose group combinations in the model fit of EC_PL_1. The top labels indicate the morphine dose in mg/kg. The side labels indicate the absence or presence of Pgp inhibitor GF120918. The columns and row indicated with (all) display the combination of all dose groups or all Pgp inhibitor groups, respectively. The colors are for visual distinction of the animal IDs.


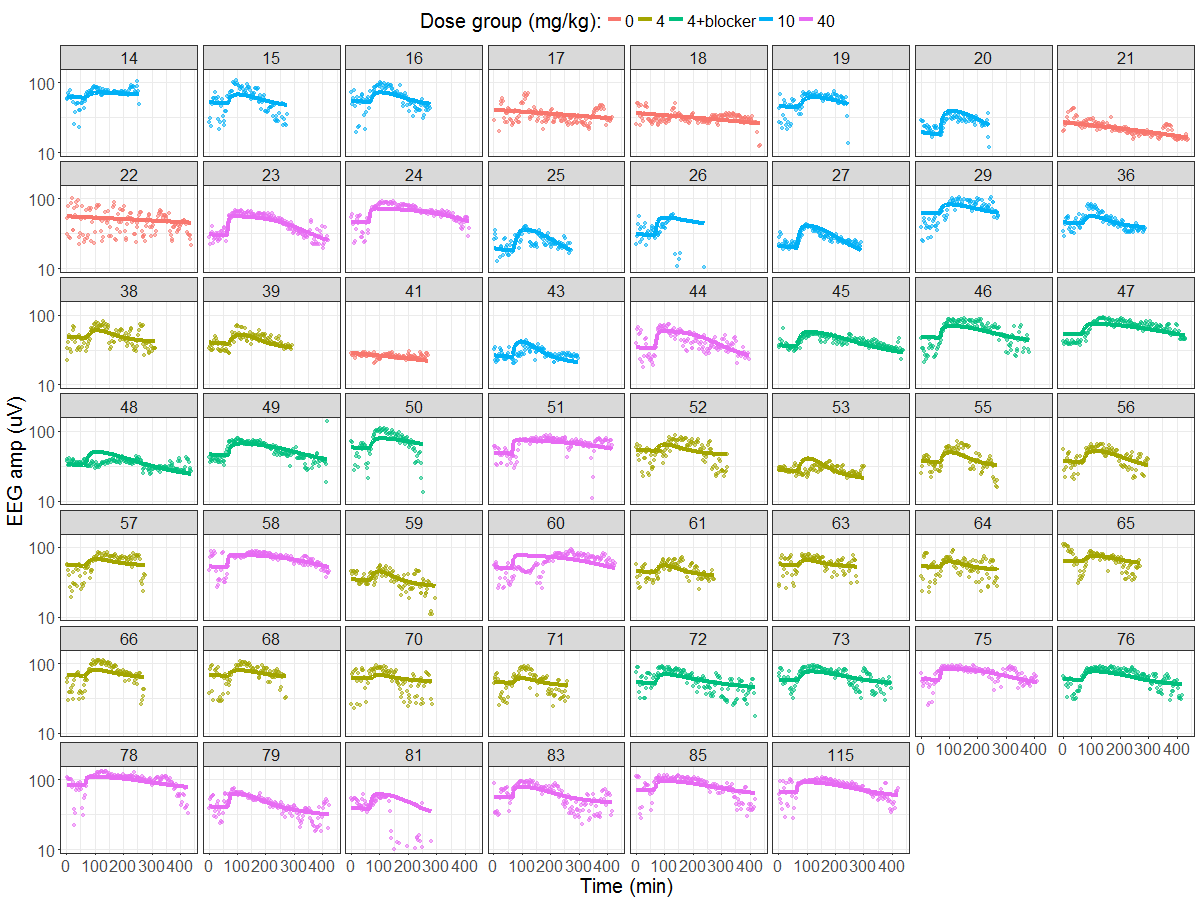


Figure S 9. Individual model fits of model EC_PL_1 to the EEG data. The colors represent the different dose groups. Dots represent the observations, lines the model predictions.

No inhibitor

No inhibitor

No inhibitor


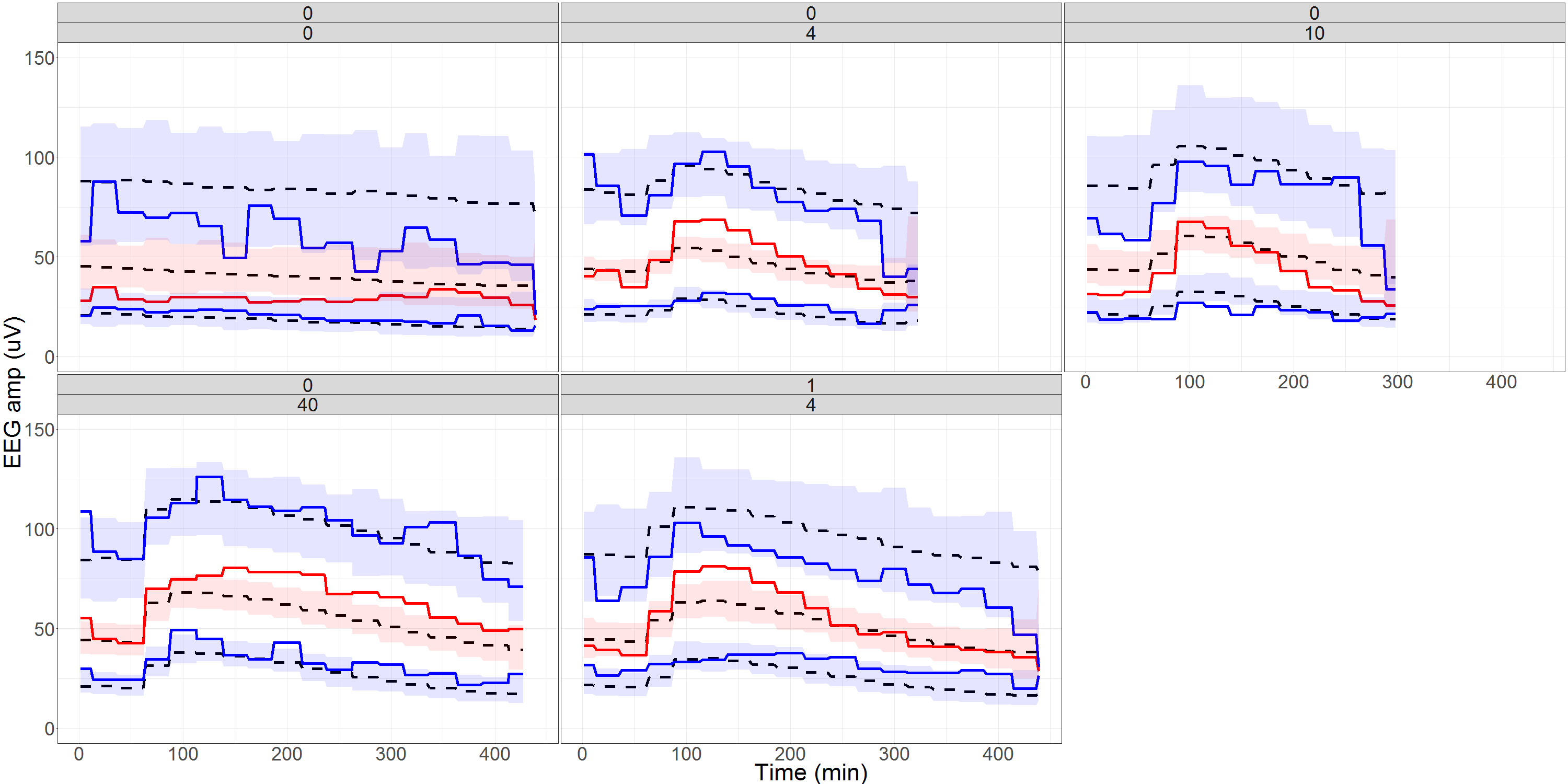


4 mg/kg

10 mg/kg

0 mg/kg

4 mg/kg

With inhibitor

No inhibitor

40 mg/kg

Figure S 10. Visual predictive check of the model fit of model EC_PL_1. The upper labels indicate the absence or presence of Pgp inhibitor GF120918. The lower labels indicate the morphine dose in mg/kg. The solid lines represent the observed 5%, 50% and 95% quantiles of the data. The dashed lines represent the median of the 5%, 50% and 95% quantiles of the simulated datasets. The shaded areas represent the 5%-95% percent interval of the 5%, 50% and 95% quantiles of the simulated datasets.

**Model equations and VPC for model EC-TB_PL_1**

The model equations for the EC-TB_PL_ model are provided in equations 13-15. In these equations, *A_EFF_* and *V_EFF_* refer to the amount and volume of the effect compartment, respectively. *A_RL_* and *V_RL_* refer to the amount and volume of the drug-target complex compartment, respectively. *A_Rtot_* and *V_Rtot_* refer to the amount and volume of the bound plus unbound target compartment, respectively. The rate constants *k_e0_* and *k_off_* are first order rate constants of distribution and dissociation, respectively. *k_on_* is the second order association rate constant. *E_0_* is the baseline EEG amplitude, *slope* is the linear decline of the EEG amplitude per time unit, independent of the drug effect and *Emax* is the maximal drug effect. *A_EFF_*/*V_EFF_* and *A_RL_*/*V_RL_* are 0 at time t = 0.

No inhibitor

4 mg/kg

No inhibitor

10 mg/kg

No inhibitor


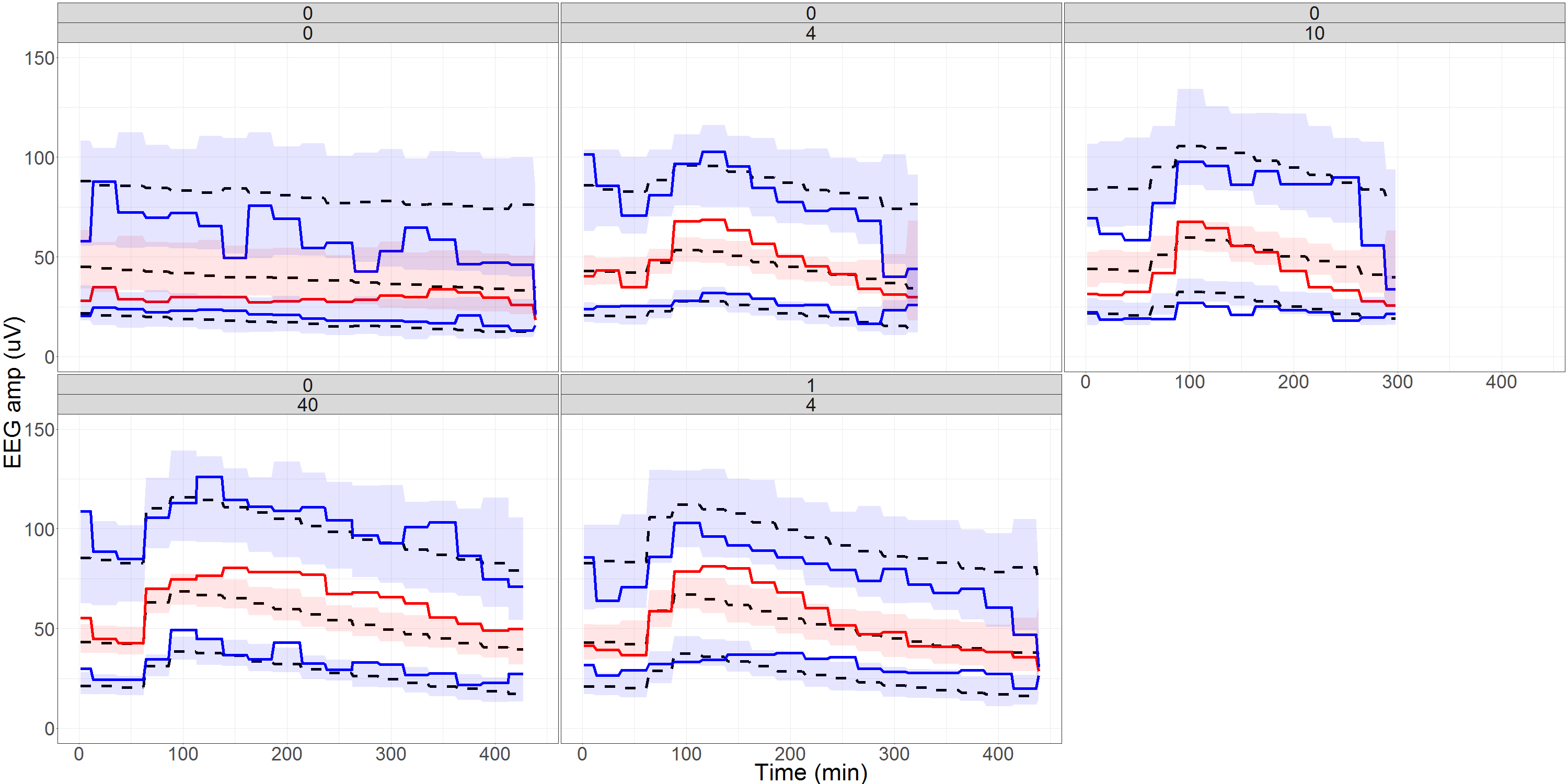


40 mg/kg

4 mg/kg

With inhibitor

No inhibitor

0 mg/kg

Figure S 11. Visual predictive check of the model fit of model EC-TB_PL_1. The upper labels indicate the absence or presence of Pgp inhibitor GF120918. The lower labels indicate the morphine dose in mg/kg. The solid lines represent the observed 5%, 50% and 95% quantiles of the data. The dashed lines represent the median of the 5%, 50% and 95% quantiles of the simulated datasets.

**Model equations and VPC for model TB_PL_4**

The model equations for the combined TB_PL_ model are provided in equations 16 and 17. In these equations, *A_RL_* and *V_RL_* refer to the amount and volume of the drug-target complex compartment, respectively. *A_Rtot_* and *V_Rtot_* refer to the amount and volume of the bound plus unbound target compartment, respectively. The rate constants *k_off_* is the first order rate constants of drug-target dissociation. *k_on_* is the second order association rate constant. *E_0_* is the baseline EEG amplitude, *slope* is the linear decline of the EEG amplitude per time unit, independent of the drug effect and *Emax* is the maximal drug effect. *A_RL_*/*V_RL_* is 0 at time t = 0.

$\frac{d\frac{A_{RL}}{V_{RL}}}{dt}= k_{on} \cdot\frac{A_{c}}{V_{C}} \cdot\left( Rtot- \frac{A_{RL}}{V_{RL}} \right)-k_{off} \cdot\frac{A_{RL}}{V_{RL}}$

$Effect \left( EEG amplitude \right)= E_{0}+slope*t + \frac{E_{max} \cdot\frac{A_{RL}}{V_{RL}}}{\frac{A_{Rtot}}{V_{Rtot}}}$

No inhibitor

No inhibitor

10 mg/kg

No inhibitor

4 mg/kg


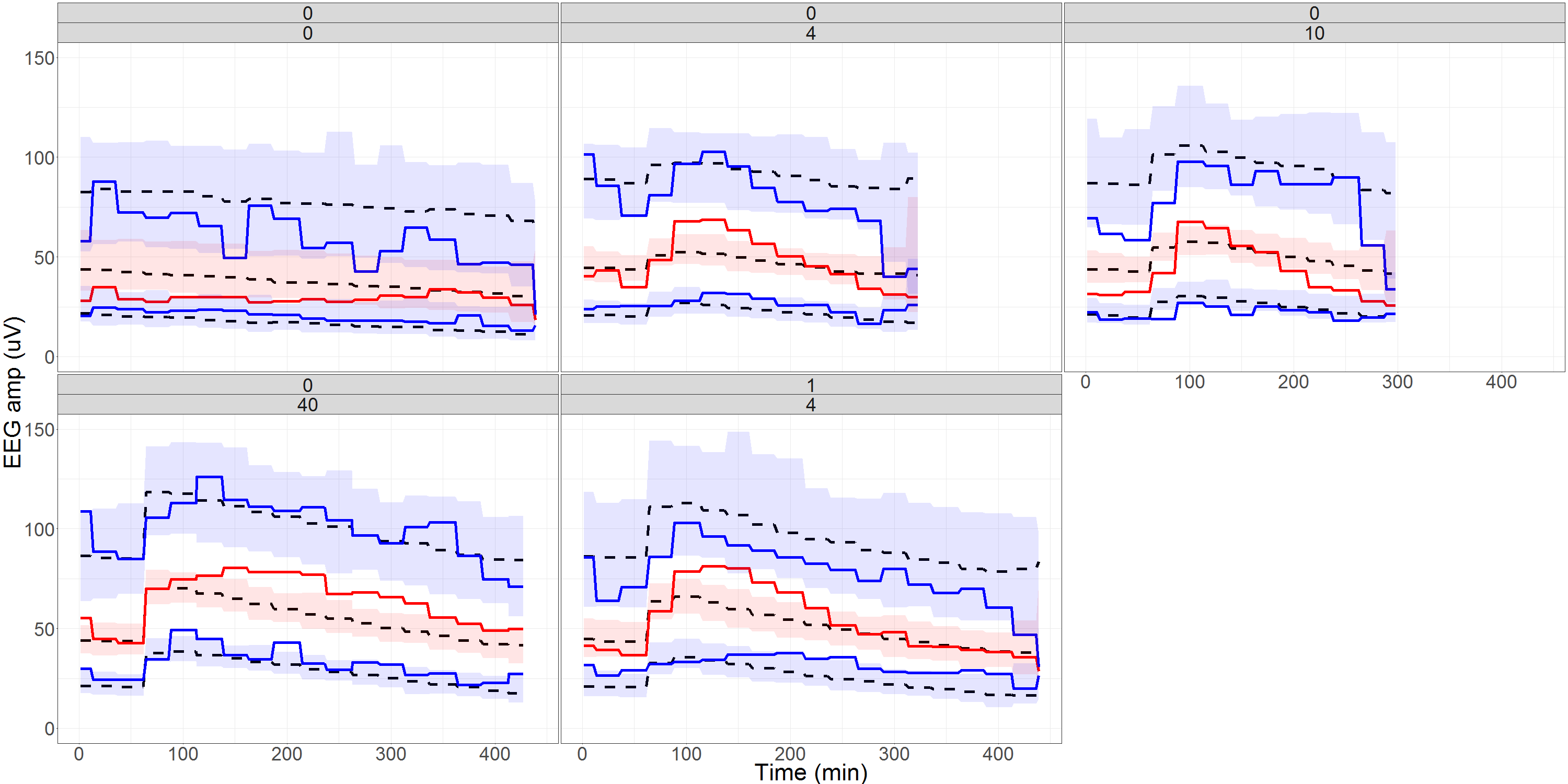


No inhibitor

40 mg/kg

4 mg/kg

With inhibitor

0 mg/kg

Figure S 12. Visual predictive check of the model fit of model TB_PL_4. The upper labels indicate the absence or presence of Pgp inhibitor GF120918. The lower labels indicate the morphine dose in mg/kg. The solid lines represent the observed 5%, 50% and 95% quantiles of the data. The dashed lines represent the median of the 5%, 50% and 95% quantiles of the simulated datasets.

## Supplement S 2. Dose-dependency of Tmax_TO_ in a TB_PL_.

To obtain a better understanding of the influence of dose on the Tmax_TO_ in a TB_PL_ model, some of the underlying simulations for Figure 5 are shown in this section. In Figure S 13, the simulation with the lowest values of *k_on_* and *k_off_* is showing that in this situation, the Tmax_TO_ has a high value, but also that there is no difference between the two doses. This can be understood by comparing the rate of equilibration in a situation with a constant ligand concentration with the rate of elimination.

The rate of equilibration (*k_obs_*) for a constant ligand concentration [*L*] can be calculated by equation 1 [1]:

*k_obs_* = *kon* * [*L*] + *koff* (1)

Since the ligand concentration in our simulations is normalized for the value of *K_D_*, equation 1 can be rewritten as equation 2, in which c is the ratio [*L*]/*K_D_*:

*k_obs_* = *kon* * *c* * *koff*/*kon* + *koff* = *koff**(*c* + 1) (2)

From equation 2, it can be observed that a low value of *k_off_* leads to slow equilibration, unless the ligand concentration is much higher than the affinity. If the equilibration rate is slow, the Tmax_TO_ is mainly determined by the elimination rate constant, which is independent on the dose/ligand concentration. Thus, a low value of *k_off_* gives similar Tmax_TO_ values for different doses, as confirmed in Figure S 13.


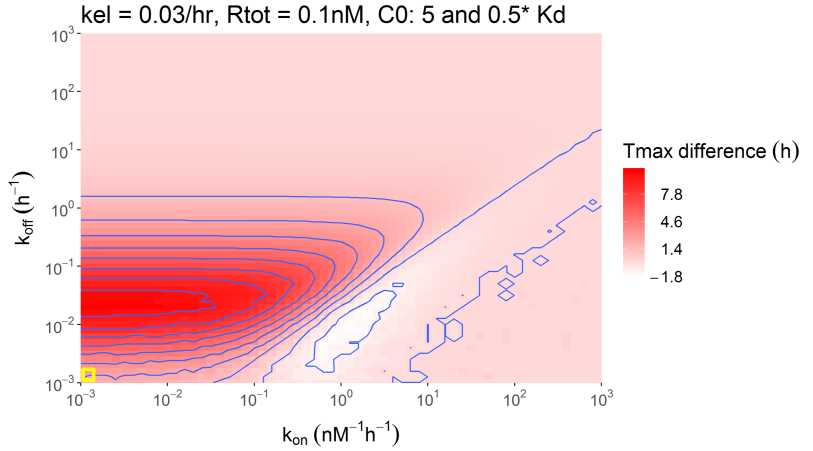

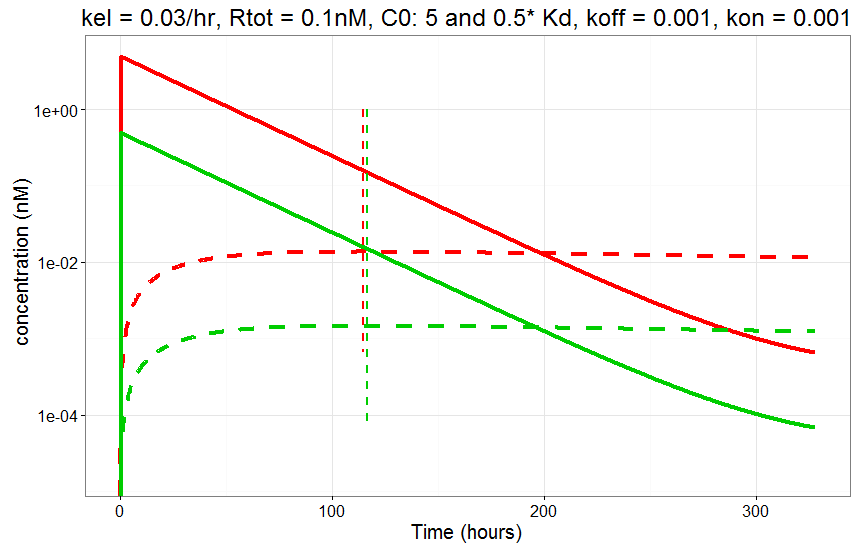


Figure S 13. Simulation of drug target binding for two different doses. The solid lines indicate plasma concentrations for the high (red) dose and the low (green) dose. The dashed lines indicate target-bound drug concentrations. The vertical dotted lines indicate the time point of the maximal target-bound concentration for each dose. In this simulation, the elimination rate constant *k_el_* was 0.03/hr and the target concentration was 0.1 nM. The initial concentrations for the high and the low dose corresponded to 5 and 0.5 times the *K_D_*, respectively. The *k_on_* and *k_off_* values were 0.001 nM^-1^ h^-1^  and 0.001 h^-1^, respectively, representing the pixel of Figure 5 that is indicated with the yellow square in the right panel.

A high value of *k_off_* gives rise to fast equilibration and a significant influence of the dose on the equilibration time because equilibration is now much faster than elimination, and thus determining the Tmax_TO_. However, because of the fast equilibration, the decrease in Tmax_TO_ with increasing doses is difficult to detect because all Tmax_TO_ values are low, and the absolute difference is low as well, as illustrated in Figure S 14.


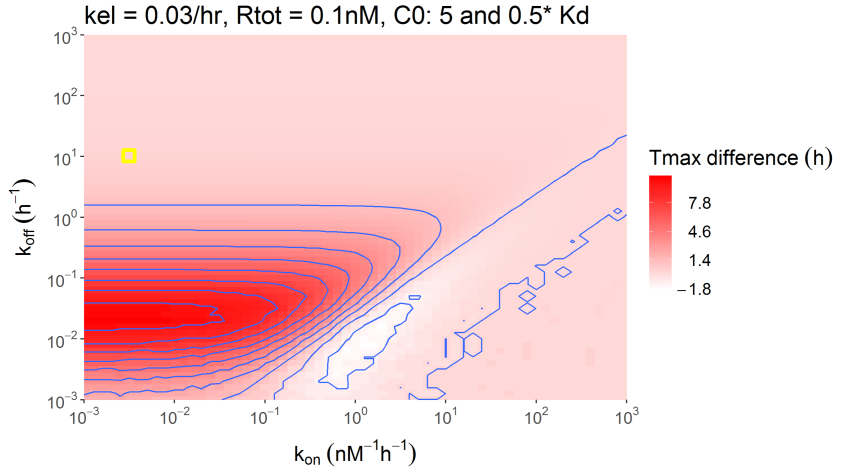

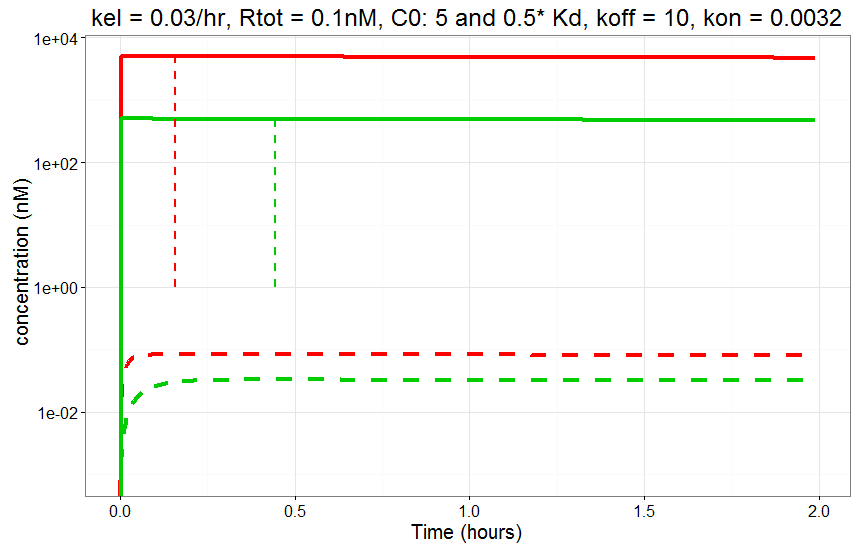


Figure S 14. Simulation of drug target binding for two different doses. The solid lines indicate plasma concentrations for the high (red) dose and the low (green) dose. The dashed lines indicate target-bound drug concentrations. The vertical dotted lines indicate the time point of the maximal target-bound concentration for each dose. In this simulation, the elimination rate constant k_el_ was 0.03/hr and the target concentration was 0.1 nM. The initial concentrations for the high and the low dose corresponded to 5 and 0.5 times the *K_D_*, respectively. The *k_on_* and *k_off_* values were 0.0032 nM^-1^ h^-1^ and 10 h^-1^, respectively, representing the pixel of Figure 5 that is indicated with the yellow square in the right panel.

A low value of the *K_D_* (and therefore a low dose) will also lead to a difference in Tmax_TO_ which is negligibly small or sometimes even negative (i.e. the highest dose leads to the highest Tmax_TO_ value). In this area the assumption of a constant ligand concentration does not hold anymore, even when there is no elimination of the drug. This is caused by the depletion of ligand as a result of drug-target binding. When the ligand concentration is much lower than the target concentration, the equilibration rate can now be approximated by assuming the target concentration (*R_tot_*) is constant, according to equation 3:

*k_obs_* = *k_on_* * [*R_tot_*] + *k_off_* (3)

From equation 3, it should be observed that there is no influence of the ligand concentration any more, and therefore the dose does not influence the Tmax_TO_ anymore. The small band in Figure 5 where the difference in Tmax_TO_ values is negative can be explained by the situation where the lowest dose has the same target concentration and drug concentration. In this case, both the target and the drug concentration decline upon drug-target binding and equilibration is twice as fast compared to the situation with a constant target or ligand concentration. This can make the equilibration of the lowest dose faster than that of the highest dose. An example of such a situation is shown Figure S 15.


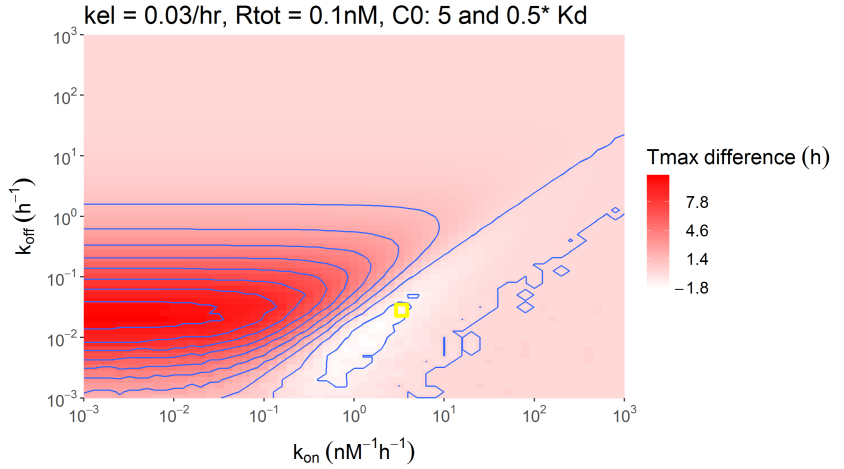

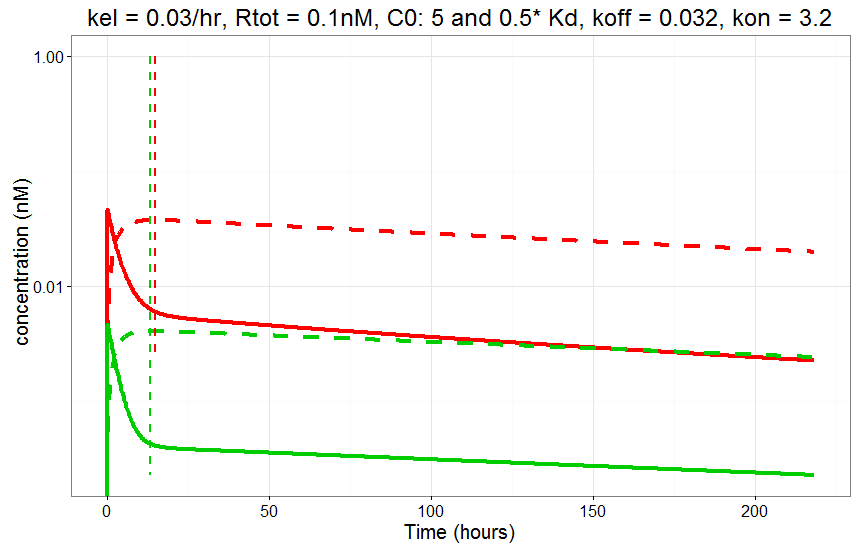


Figure S 15. Simulation of drug target binding for two different doses. The solid lines indicate plasma concentrations for the high (red) dose and the low (green) dose. The dashed lines indicate target-bound drug concentrations. The vertical dotted lines indicate the time point of the maximal target-bound concentration for each dose. In this simulation, the elimination rate constant k_el_ was 0.03/hr and the target concentration was 0.1 nM. The initial concentrations for the high and the low dose corresponded to 5 and 0.5 times the *K_D_*, respectively. The *k_on_* and *k_off_* values were 3.2 nM^-1^ h^-1^ and 0.032 h^-1^, respectively, representing the pixel of Figure 5 that is indicated with the yellow square in the right panel.

To observe a change in Tmax_TO_, it follows from the previous examples that the value of k_off_ should be low enough to make the change in Tmax_TO_ observable, but it should not be so low that the elimination of the drug determines the Tmax_TO_. Moreover, the initial concentration of the drug should not be lower than the target concentration. An example of such a situation is given in Figure S 16. Additionaly, the lines *k_off_* = *k_el_*/(*c*+1) and *K_D_* = *Rtot*/(*c*+1) align reasonably well with the middle and the diagonal end of the area where Tmax_TO_ is most significant, where *c* represents the initial concentration/*K_D_* ratio for the lowest dose as shown in Figure S 17.


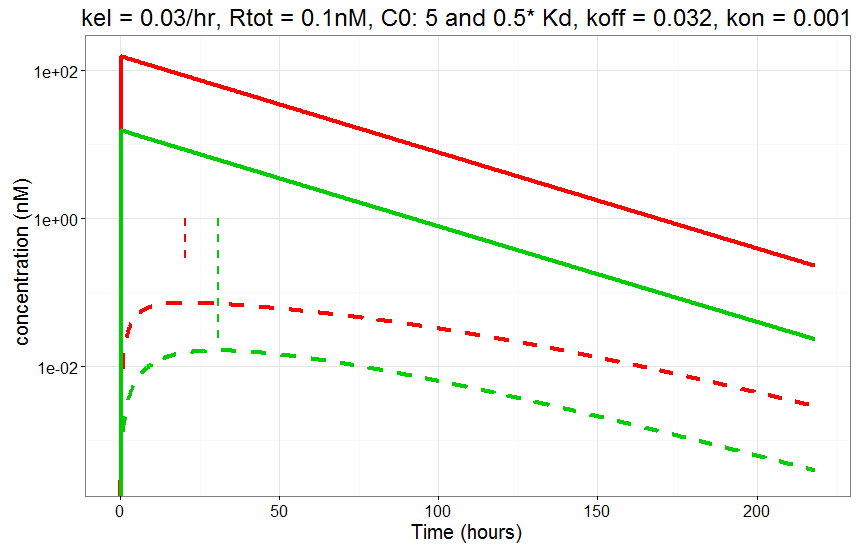

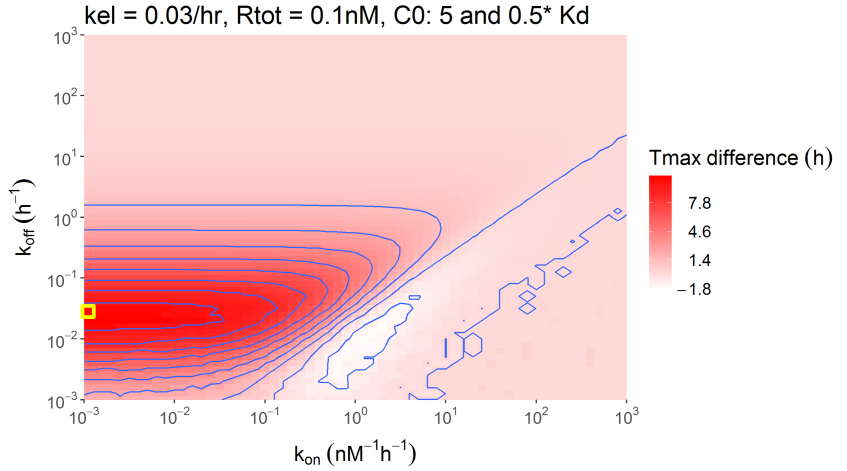


Figure S 16. Simulation of drug target binding for two different doses. The solid lines indicate plasma concentrations for the high (red) dose and the low (green) dose. The dashed lines indicate target-bound drug concentrations. The vertical dotted lines indicate the time point of the maximal target-bound concentration for each dose. In this simulation, the elimination rate constant *k_el_* was 0.03/hr and the target concentration was 0.1 nM. The initial concentrations for the high and the low dose corresponded to 5 and 0.5 times the *K_D_*, respectively. The *k_on_* and *k_off_* values were 0.001 nM^-1^ h^-1^ and 0.032 h^-1^, respectively, representing the pixel of Figure 5 that is indicated with the yellow square in the right panel.


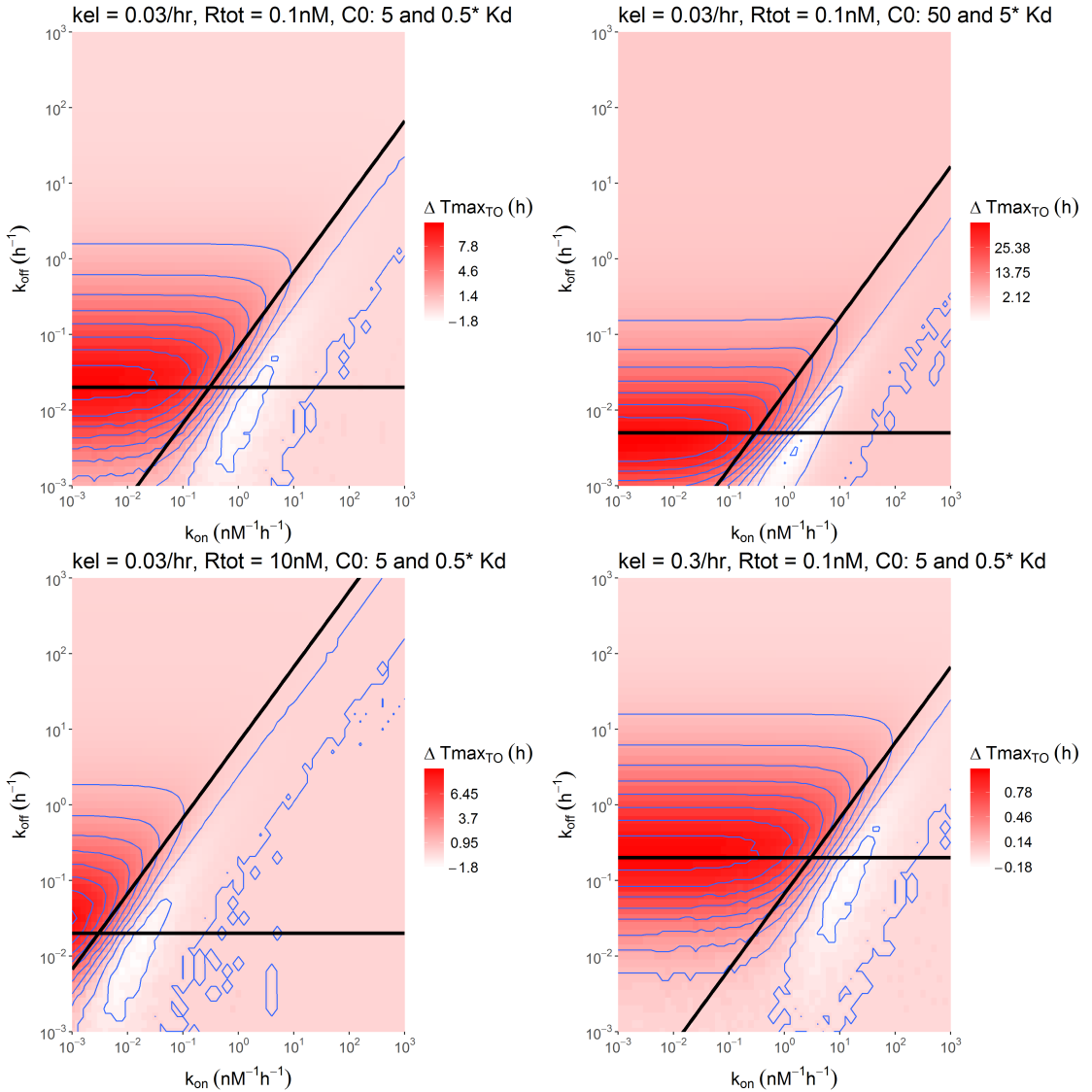


Figure S 17. Overview of the ∆Tmax_TO_ that was observed in the simulations as a result of the change in the affinity-normalized dose for different combinations of parameter values as indicated above the panels. Each pixel represents a single simulation in which the k_off_ and k_on_ value correspond to the position on the y-axis and the x-axis, respectively, and the color represents the observed shift in Tmax_TO_ in that simulation. All panels vary only one parameter compared to the upper left panel. The horizontal and diagonal lines represent the equations *k_off_* = *k_el_*/(*c*+1) and *K_D_* = *R_tot_*/(*c*+1), respectively, where *c* represents the initial concentration/*K_D_* ratio for the lowest dose.

**Supplementary references**

1. Tummino PJ, Copeland RA (2008) Residence time of receptor-ligand complexes and its effect on biological function. biochemistry 47:5481–92.

# Supplement S 3. Asymptotic analysis of and its dependency on the dose.

## 1. One compartment model with drug-target binding

The model for drug-target binding is given by

where

• is the drug concentration,

• is the **free** receptor concentration,

• is the concentration of bound complex of and : ,

• is the rate constant at which binds to free receptors,

• is the rate constant at which unbinds,

• is the elimination rate constant.

Now, we use that the **total** receptor concentration is described by so that , and hence, . Then, after substituting this expression for , the system becomes

and hence,

(1.1)

We study this system together with the initial conditions and .

The aim of this analysis is to determine the value of where attains a maximum for general . We denote this maximum by . Furthermore, the interest is to determine the difference in for two (different) values of . More specifically for and where , we want to determine .

## 2. Rescaling the system

In order to be able to analyse system (1.1), we rescale it by using the fact that both and can maximally reach certain concentrations. From the initial conditions it follows that the drug is limited by drug dose . Also, the bound complex is limited by the total receptor concentration . This suggests to rescale with and with , and therefore, set and . Then system (1.1) becomes

(2.1)

with and . In this system corresponds to and to .

Next, we study system (2.1) in different parameter regions and determine the value of for which attains a maximum.We use the different sets of coefficients present in system (2.1) to determine the various regions. In these regions, we use asymptotic analysis to determine an asymptotic expansion for from which we determine the leading order of .

To define the regions, we look at the groups of parameters present in system (2.1) and set them to be equal. This gives us the following lines

(2.2)

In the following analysis, we assume that is of order 1, hence , then we define various regions

Note that since , not all the lines in (2.2) are needed when defining these regions. On the other hand, when *c* >> 1, the different lines are essential.

*
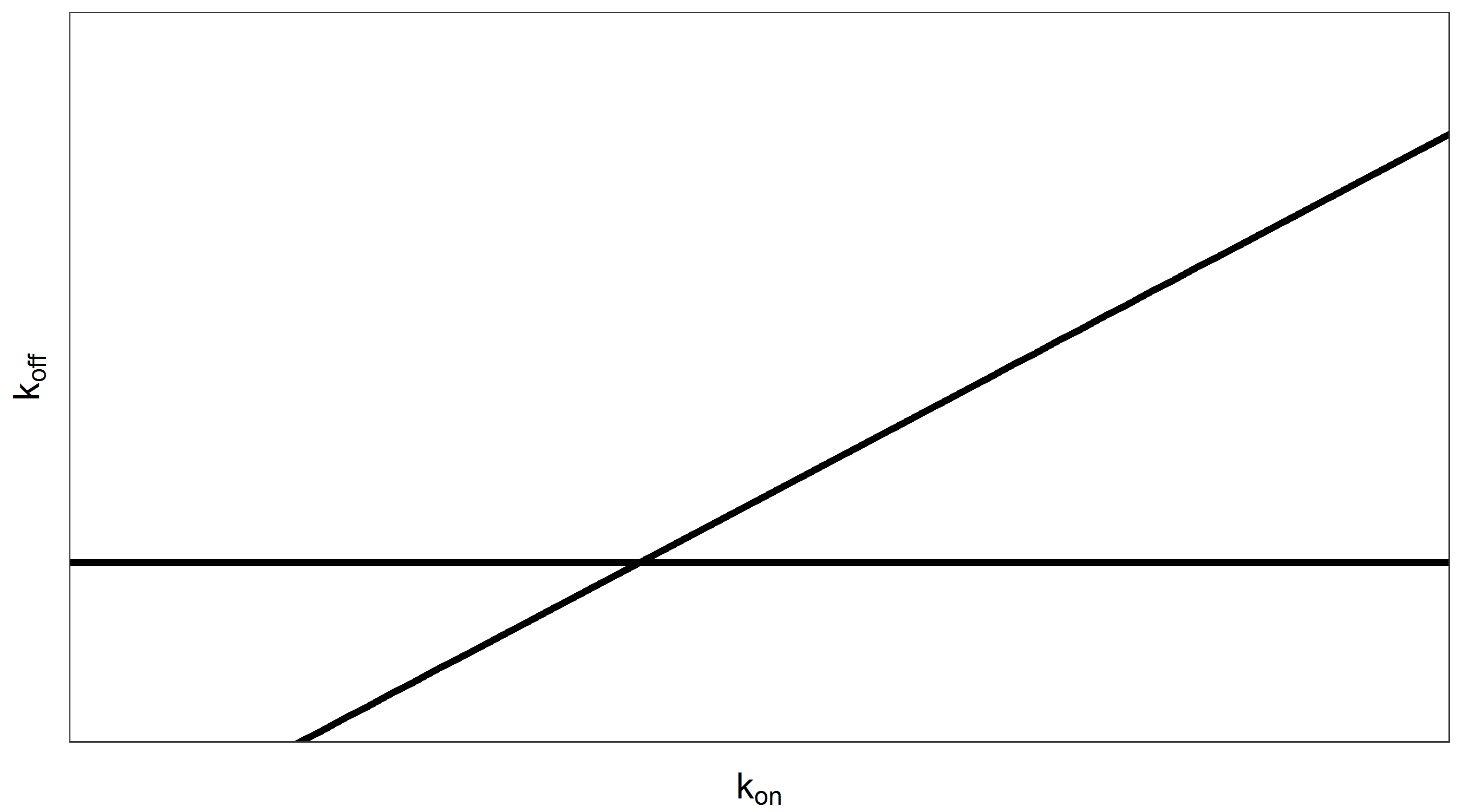
*

*Figure S18: Sketch of the different regions in the -plane and the lines and for .*

In these regions, we can find an asymptotic expression for and determine the leading order expression for . Here, we summarise the results.

In region , we find that must satisfy an implicit relation depending on the different parameters. We introduce then , where must satisfy

(2.3)

In the other regions we can determine the leading order of explicitly, this yields:

Note that we denote with the natural logarithm, .

Hence, we find that when (in regions III and V) that  **does not depend on** , and therefore, is independent of the dosis, to leading order. For (in regions II and IV), we find from the above expressions that  **is small**, and so the dependence on the dose does also not play a role.

Note that the above results are not true for . We briefly study that case in section 5.

To show how we obtain the above results, we give the details of the asymptotic analysis in two of the regions in the next sections.

## 3. The analysis in region III

We choose the parameters to lie in region III such that and . Then, we rescale time as in this region and system (2.1) becomes

(3.1)

where and . From the choice of the relation between the parameters, we find that . Now, we assume the following asymptotic expansions for and

And, from the initial conditions for system (2.1), it follows that , for all .

In the following we assume that and are not of the same order. Next, we substitute the above expansions into system (3.1), collect terms at different orders and solve the corresponding equations at each level.

At , we find that

This can be solved and, together with the initial conditions, this leads to and .

Next, at we obtain

Together with the initial conditions, this gives and . Since this does not attain a maximum, we need to determine higher order terms in the expansion of .

Then, at we find

which yields . It turns out we don’t need to determine so we refrain from giving that here.

At we obtain

From this, we find

Since we do not need for further analysis, we also do not give that here.

Now, collecting the various terms, we find that

to leading order. Using this expression, we can obtain a leading order expression for . Differentiating we find

Setting this expression to zero, we can find from a balance between the first and the last term. Hence, we set which leads to

Rescaling back to original variables and parameters, we obtain

## 4. The analysis in region V

In this section, we choose the parameters to lie in region V such that and . We rescale time as in this region and system (2.1) becomes

(4.1)

where and . From the choice of the relation between the parameters, we find that . Now, we assume the following asymptotic expansions for and

From the initial conditions for system (2.1), it follows that , for all .

In the following we assume that and are not of the same order. Next, we substitute the above expansions into system (4.1), collect terms at different orders and solve the corresponding equations at each level.

At we find that

This can be solved and, together with the initial conditions, this leads to and .

Next, at we find

which yields . Solving for leads to

At we obtain

Together with the initial conditions, this gives . We do not give since we will not need it in the further analysis. Again, does not attain maximum, and therefore, we need higher order terms in the expansion of .

At we find that and so we need to go to where

Hence,

and the expansion for reads

to leading order. Differentiating leads to

which becomes zero when

Rescaling back to original variables and parameters, we obtain

## 5. The case when .

Next, we briefly look at the case when . Then, the results are different from before. One essential difference is that the regions now depend on where .

We will only give results for region . Note that this region shifts down in the -plane compared to before.

We do still find that must satisfy an implicit relation depending on the different parameters. We find that , where must satisfy

(5.1)

and .
